# Supplementary material for: Distinguishing moral hazard from access for high-cost healthcare under insurance
Source: PLoS One. 2020 Apr 17;15(4):e0231768. doi: 10.1371/journal.pone.0231768 (PMC7164657; doi:10.1371/journal.pone.0231768)
Supplement: S1 Table — (DOCX) [file pone.0231768.s001.docx]

**Table S1. Experiment 1 -- Demographics Split by Insurance Type Manipulation**

|  | **Whole**  **Sample** | **No  Insurance** | **Traditional Insurance** | **Indemnity Insurance** |
| --- | --- | --- | --- | --- |
| **Size** | 613 | 204 | 207 | 202 |
|  |  |  |  |  |
| **Age** |  |  |  |  |
| 25 or younger | 13.09% | 8.82% | 17.56% | 12.87% |
| 26 - 35 | 42.88% | 42.16% | 40.00% | 46.53% |
| 36 - 45 | 22.09% | 24.51% | 22.44% | 19.31% |
| 46 or order | 21.93% | 24.51% | 20.00% | 21.29% |
|  |  |  |  |  |
| **Education** |  |  |  |  |
| Less than high school degree | 0.65% | 0.49% | 0.48% | 0.99% |
| High school graduate or equivalent | 10.44% | 11.27% | 10.63% | 9.41% |
| Some college but no degree | 29.85% | 28.43% | 32.37% | 28.71% |
| Associate degree in college | 12.72% | 14.22% | 9.66% | 14.36% |
| Bachelor's degree in college | 33.93% | 34.80% | 34.30% | 32.67% |
| Master's degree | 9.46% | 7.84% | 10.63% | 9.90% |
| Doctoral degree | 0.98% | 0.98% | 0.48% | 1.49% |
| Professional degree (JD, MD) | 1.96% | 1.96% | 1.45% | 2.48% |
|  |  |  |  |  |
| **Race** |  |  |  |  |
| White | 72.01% | 75.00% | 69.57% | 71.50% |
| Black or African American | 8.18% | 7.35% | 8.70% | 8.50% |
| American Indian or Alaska Native | 0.82% | 0.00% | 1.93% | 0.50% |
| Asian | 10.15% | 11.27% | 8.21% | 11.00% |
| Other | 3.11% | 0.49% | 4.35% | 4.50% |
| Mixed | 5.73% | 5.88% | 7.25% | 4.00% |
|  |  |  |  |  |
| **Sex** |  |  |  |  |
| Male | 52.87% | 51.74% | 52.66% | 54.23% |
| Female | 47.13% | 48.26% | 47.34% | 45.77% |
|  |  |  |  |  |
| **Before-Tax Income** |  |  |  |  |
| Less than $10,000 | 8.69% | 5.42% | 11.11% | 9.50% |
| $10,000 to $49,999 | 49.35% | 52.71% | 47.35% | 48.00% |
| $50,000 to $99,999 | 31.96% | 31.52% | 30.92% | 33.50% |
| $100,000 or more | 10.00% | 10.34% | 10.63% | 9.00% |
|  |  |  |  |  |
| **Spending Power** |  |  |  |  |
| Less than $10,000 | 51.16% | 55.94% | 47.80% | 49.75% |
| $10,000 to $49,999 | 27.57% | 24.26% | 30.73% | 27.64% |
| $50,000 to $99,999 | 10.07% | 10.40% | 10.24% | 9.55% |
| $100,000 or more | 11.23% | 9.41% | 11.22% | 13.06% |
