# Supplementary material for: Distinguishing moral hazard from access for high-cost healthcare under insurance
Source: PLoS One. 2020 Apr 17;15(4):e0231768. doi: 10.1371/journal.pone.0231768 (PMC7164657; doi:10.1371/journal.pone.0231768)
Supplement: S2 Table — (DOCX) [file pone.0231768.s002.docx]

**Table S2. Experiment 2 -- Demographics Split by Insurance Type Manipulation**

|  | **Whole Sample** | **No Insurance** | **Traditional Insurance** | **Indemnity Insurance** |
| --- | --- | --- | --- | --- |
| **Size** | 2356 | 791 | 858 | 707 |
|  |  |  |  |  |
| **Age** |  |  |  |  |
| 30 or younger | 19.16% | 19.48% | 20.03% | 17.75% |
| 30 – 39 | 20.45% | 22.09% | 19.00% | 20.37% |
| 40 – 49 | 15.82% | 15.23% | 16.30% | 15.90% |
| 50 – 59 | 18.51% | 17.97% | 20.54% | 16.67% |
| 60 – 69 | 17.12% | 16.46% | 16.05% | 19.14% |
| 70 or older | 8.95% | 8.78% | 8.09% | 10.19% |
|  |  |  |  |  |
| **Education** |  |  |  |  |
| Less than high school degree | 2.63% | 3.41% | 2.56% | 1.84% |
| High school graduate or equivalent | 17.06% | 18.20% | 17.13% | 15.70% |
| Some college but no degree | 24.83% | 24.78% | 26.22% | 23.20% |
| Associate degree in college | 10.57% | 9.86% | 10.84% | 11.03% |
| Bachelor's degree in college | 28.10% | 26.30% | 28.21% | 29.99% |
| Master's degree | 12.52% | 13.78% | 10.61% | 13.44% |
| Doctoral degree | 1.70% | 1.52% | 1.75% | 1.84% |
| Professional degree (JD, MD) | 2.59% | 2.15% | 2.68% | 2.97% |
|  |  |  |  |  |
| **Hispanic** |  |  |  |  |
| Non-Hispanic | 91.77% | 91.53% | 92.07% | 91.65% |
| Hispanic | 8.23% | 8.47% | 7.93% | 8.35% |
|  |  |  |  |  |
| **Race** |  |  |  |  |
| White | 80.31% | 81.29% | 79.37% | 80.34% |
| Black | 5.98% | 5.56% | 5.94% | 6.51% |
| Native American | 0.47% | 0.51% | 0.35% | 0.57% |
| Asian | 6.24% | 5.18% | 7.11% | 6.36% |
| Other | 2.08% | 2.15% | 2.45% | 1.56% |
| Mixed | 4.92% | 5.31% | 4.78% | 4.67% |
|  |  |  |  |  |
| **Sex** |  |  |  |  |
| Male | 47.88% | 46.65% | 48.95% | 47.95% |
| Female | 52.12% | 53.35% | 51.05% | 52.05% |
|  |  |  |  |  |
| **Income** |  |  |  |  |
| Less than $10,000 | 5.60% | 5.44% | 6.29% | 4.95% |
| $10,000 to $19,999 | 9.68% | 8.98% | 10.61% | 9.34% |
| $20,000 to $29,999 | 9.42% | 9.36% | 9.79% | 9.05% |
| $30,000 to $39,999 | 10.27% | 11.50% | 9.67% | 9.62% |
| $40,000 to $49,999 | 7.85% | 8.09% | 8.97% | 6.22% |
| $50,000 to $59,999 | 7.26% | 8.22% | 6.76% | 6.79% |
| $60,000 to $69,999 | 6.20% | 7.33% | 4.90% | 6.51% |
| $70,000 to $79,999 | 6.66% | 5.94% | 7.11% | 6.93% |
| $80,000 to $89,999 | 4.92% | 4.42% | 5.83% | 4.38% |
| $90,000 to $99,999 | 5.09% | 4.93% | 4.90% | 5.52% |
| $100,000 to $149,999 | 14.43% | 14.79% | 12.24% | 16.69% |
| $150,000 or more | 12.61% | 11.00% | 12.94% | 14.00% |
|  |  |  |  |  |
| **Purchasing Power (N = 2048)** |  |  |  |  |
| Less than $500 | 23.97% | 22.81% | 24.06% | 25.16% |
| from $500 to $1000 | 43.85% | 44.44% | 41.58% | 45.94% |
| from $1000 to $1500 | 2.73% | 2.63% | 2.94% | 2.60% |
| from $1500 to $2500 | 5.27% | 4.39% | 6.55% | 4.71% |
| from $2500 to $3500 | 2.78% | 3.07% | 3.07% | 2.11% |
| from $3500 to $5500 | 8.89% | 9.94% | 8.29% | 8.44% |
| from $5500 to $7500 | 11.08% | 10.96% | 12.17% | 9.90% |
| $7500 or more | 1.42% | 1.75% | 1.34% | 1.14% |
|  |  |  |  |  |
| **Vignette** |  |  |  |  |
| Cancer: Adenocarcinoma of the colon | 12.22% | 10.75% | 15.03% | 10.47% |
| Cancer: Non-small-cell lung cancer | 13.07% | 12.39% | 12.59% | 14.43% |
| Cardiovascular: Coronary Artery Disease | 11.50% | 12.77% | 10.84% | 10.89% |
| Gastrointestinal Disease: GERD | 12.48% | 12.77% | 12.00% | 12.73% |
| Age-Related Macular Degeneration (AMD) | 12.69% | 11.88% | 13.17% | 13.01% |
| Skin/Autoimmune disease: Psoriasis | 12.10% | 13.65% | 11.66% | 10.89% |
| Orthopedic Disease: Degenerative Arthritis | 13.41% | 13.53% | 12.47% | 14.43% |
| Neurologic Disease: Degenerative Lower Spine Disease | 12.52% | 12.26% | 12.24% | 13.15% |
