# Supplementary material for: Distinguishing moral hazard from access for high-cost healthcare under insurance
Source: PLoS One. 2020 Apr 17;15(4):e0231768. doi: 10.1371/journal.pone.0231768 (PMC7164657; doi:10.1371/journal.pone.0231768)
Supplement: S3 Table — (DOCX) [file pone.0231768.s003.docx]

**Table S3: Online Convenience Sample Experiment (Amazon.com) – Linear Probability Models on Intent to Consume Treatment**

**Panel A: No Insurance v. Indemnity Insurance**

|  | Full Sample | | Impossibility Screened | Manipulation Screened | Both Checks Screened |
| --- | --- | --- | --- | --- | --- |
| Indemnity | 0.222*** | 0.225*** | 0.393*** | 0.266*** | 0.400*** |
| (Access) | (0.064) | (0.067) | (0.061) | (0.070) | (0.062) |
| Value of Healthcare | 0.015 | 0.012 | 0.033 | 0.032 | 0.032 |
|  | (0.064) | (0.066) | (0.063) | (0.067) | (0.062) |
| Indemnity X Value | 0.172 | 0.165 | 0.152 | 0.140 | 0.157 |
|  | (0.090) | (0.094) | (0.085) | (0.100) | (0.088) |
| Constant | 0.208*** | 0.146 | -0.253 | -0.022 | -0.407* |
|  | (0.045) | (0.202) | (0.191) | (0.213) | (0.197) |
| Controls | NO | YES | YES | YES | YES |
| R-squared | 0.121 | 0.128 | 0.292 | 0.154 | 0.325 |
| N | 406 | 390 | 357 | 331 | 305 |

**Panel B: Traditional Insurance vs. Indemnity Insurance**

|  | Full Sample | | Manipulation Screened |
| --- | --- | --- | --- |
| Traditional Insurance | 0.0367 | 0.0789 | 0.1006 |
| (Moral Hazard) | (0.0679) | (0.0697) | (0.075) |
| Value of Healthcare | 0.1877*** | 0.2046*** | 0.2035** |
|  | (0.0683) | (0.0704) | (0.0797) |
| Traditional Insurance X Value | 0.0614 | 0.0121 | 0.0074 |
|  | (0.0961) | (0.0991) | (0.1081) |
| Constant | 0.43*** | 0.5239 | 0.4291 |
|  | (0.0486) | (0.3515) | (0.3601) |
| Controls | NO | YES | YES |
| R-squared | 0.0537 | 0.1587 | 0.1904 |
| N | 409 | 402 | 348 |

NOTE: Standard errors shown in parentheses. “***” significant at 0.1% level; “**” significant at 1% level; “*” significant at 5% level. Manipulation screen confirms respondent attention; impossibility screen confirms ability to pay out of pocket based on self-reported assets and credit. Controls include demographics.
