# Supplementary material for: Distinguishing moral hazard from access for high-cost healthcare under insurance
Source: PLoS One. 2020 Apr 17;15(4):e0231768. doi: 10.1371/journal.pone.0231768 (PMC7164657; doi:10.1371/journal.pone.0231768)
Supplement: S4 Table — (DOCX) [file pone.0231768.s004.docx]

**Table S4: Online Census-Weighted Experiment (SSI Sample) – Regressions on Intent to Consume Treatment**

**Panel A: No Insurance v. Indemnity Insurance (Full Sample)**

|  | Linear Probability | | Logit | | Probit | |
| --- | --- | --- | --- | --- | --- | --- |
| Indemnity | 0.201*** | 0.218*** | 0.934*** | 1.095*** | 0.566*** | 0.638*** |
| (Access) | (0.035) | (0.036) | (0.165) | (0.185) | (0.099) | (0.109) |
| Value | 0.058 | 0.076* | 0.305 | 0.424* | 0.181 | 0.234* |
|  | (0.033) | (0.034) | (0.165) | (0.182) | (0.097) | (0.106) |
| Indemnity X Value | 0.138** | 0.121* | 0.492* | 0.447 | 0.317* | 0.303* |
|  | (0.048) | (0.050) | (0.225) | (0.249) | (0.136) | (0.149) |
| Constant | 0.227*** | 0.338*** | -1.227*** | -0.755 | -0.750*** | -0.452 |
|  | (0.024) | (0.095) | (0.125) | (0.473) | (0.073) | (0.285) |
| Controls | NO | YES | NO | YES | NO | YES |
| (pseudo) R-squared | 0.097 | 0.167 | 0.073 | 0.133 | 0.073 | 0.131 |
| N | 1,498 | 1,377 | 1,498 | 1,377 | 1,498 | 1,377 |

**Panel B: No Insurance v. Indemnity Insurance (Impossibility Screened)**

|  | Linear Probability | | Logit | | Probit | |
| --- | --- | --- | --- | --- | --- | --- |
| Indemnity | 0.358*** | 0.369*** | 2.308*** | 2.614*** | 1.300*** | 1.480*** |
| (Access) | (0.031) | (0.032) | (0.251) | (0.281) | (0.128) | (0.148) |
| Value | 0.021 | 0.040 | 0.285 | 0.471 | 0.141 | 0.213 |
|  | (0.032) | (0.033) | (0.296) | (0.317) | (0.146) | (0.165) |
| Indemnity X Value | 0.176*** | 0.156*** | 0.512 | 0.453 | 0.357* | 0.339 |
|  | (0.044) | (0.045) | (0.334) | (0.364) | (0.174) | (0.197) |
| Constant | 0.069** | 0.147 | -2.601*** | -2.292*** | -1.483*** | -1.314*** |
|  | (0.023) | (0.090) | (0.226) | (0.656) | (0.109) | (0.380) |
| Controls | NO | YES | NO | YES | NO | YES |
| (pseudo) R-squared | 0.254 | 0.329 | 0.221 | 0.302 | 0.221 | 0.304 |
| N | 1,345 | 1,238 | 1,345 | 1,238 | 1,345 | 1,238 |

**Panel C:   Traditional Insurance v. Indemnity Insurance**

|  | Linear Probability | | Logit | | Probit | |
| --- | --- | --- | --- | --- | --- | --- |
| Traditional Insurance | 0.064 | 0.032 | 0.257 | 0.144 | 0.161 | 0.094 |
| (Moral Hazard) | (0.035) | (0.036) | (0.143) | (0.158) | (0.089) | (0.097) |
| Value | 0.196*** | 0.194*** | 0.798*** | 0.858*** | 0.498*** | 0.532*** |
|  | (0.037) | (0.038) | (0.154) | (0.169) | (0.095) | (0.104) |
| Traditional Insurance X Value | -0.037 | -0.016 | -0.142 | -0.067 | -0.090 | -0.048 |
|  | (0.050) | (0.051) | (0.208) | (0.229) | (0.129) | (0.140) |
| Constant | 0.427*** | 0.676*** | -0.293** | 0.803 | -0.183** | 0.501 |
|  | (0.026) | (0.105) | (0.108) | (0.477) | (0.067) | (0.294) |
| Controls | NO | YES | NO | YES | NO | YES |
| (pseudo) R-squared | 0.033 | 0.107 | 0.024 | 0.082 | 0.024 | 0.081 |
| N | 1,565 | 1,427 | 1,565 | 1,427 | 1,565 | 1,427 |

 NOTE: Standard errors shown in parentheses. “***” significant at 0.1% level; “**” significant at 1% level; “*” significant at 5% level. In Experiment 2, success in manipulation check was required for respondent to submit valid data. Impossibility screen confirms ability to pay out of pocket based on self-reported assets and credit. Controls include demographics and vignette-type.
