# Supplementary material for: Distinguishing moral hazard from access for high-cost healthcare under insurance
Source: PLoS One. 2020 Apr 17;15(4):e0231768. doi: 10.1371/journal.pone.0231768 (PMC7164657; doi:10.1371/journal.pone.0231768)
Supplement: S5 Table — (DOCX) [file pone.0231768.s005.docx]

**Table S5: Cancer: Adenocarcinoma of the colon**

**Panel A: No Insurance v. Indemnity**

|  | Full Sample | | | Impossibility Screened | | |
| --- | --- | --- | --- | --- | --- | --- |
| Indemnity (Access) | 0.389*** | 0.405** | | 0.534*** | | 0.604*** |
|  | (0.109) | (0.126) | | (0.100) | | (0.115) |
| Value | 0.164 | 0.120 | | 0.061 | | 0.155 |
|  | (0.104) | (0.117) | | (0.102) | | (0.114) |
| Indemnity X Value | -0.246 | -0.183 | | -0.142 | | -0.157 |
|  | (0.152) | (0.174) | | (0.141) | | (0.161) |
| Constant | 0.205** | 0.493 | | 0.061 | | 0.159 |
|  | (0.076) | (0.257) | | (0.072) | | (0.289) |
| Controls | No | Yes | | No | | Yes |
| R-squared | 0.087 | 0.308 | | 0.246 | | 0.477 |
| N | 159 | 148 | | 140 | | 130 |
| Panel B: Indemnity v. Traditional Insurance | | | Full Sample | | | |
| Traditional Insurance (Moral Hazard) | | | -0.048 | | -0.138 | |
|  | | | (0.103) | | (0.114) | |
| Value | | | -0.081 | | -0.068 | |
|  | | | (0.116) | | (0.130) | |
| Traditional Insurance X Value | | | 0.165 | | 0.238 | |
|  | | | (0.145) | | (0.163) | |
| Constant | | | 0.595*** | | 1.163*** | |
|  | | | (0.082) | | (0.230) | |
| Controls | | | No | | Yes | |
| R-squared | | | 0.008 | | 0.215 | |
| N | | | 203 | | 187 | |
