# Supplementary material for: Distinguishing moral hazard from access for high-cost healthcare under insurance
Source: PLoS One. 2020 Apr 17;15(4):e0231768. doi: 10.1371/journal.pone.0231768 (PMC7164657; doi:10.1371/journal.pone.0231768)
Supplement: S6 Table — (DOCX) [file pone.0231768.s006.docx]

**Table S6: Cancer: Non-small-cell lung cancer**

**Panel A: No Insurance v. Indemnity**

|  | Full Sample | | Impossibility Screened | |
| --- | --- | --- | --- | --- |
| Indemnity (Access) | -0.042 | 0.036 | 0.235** | 0.251** |
|  | (0.094) | (0.104) | (0.086) | (0.095) |
| Value | -0.133 | 0.006 | 0.019 | 0.133 |
|  | (0.091) | (0.104) | (0.086) | (0.100) |
| Indemnity X Value | 0.368** | 0.275 | 0.217 | 0.147 |
|  | (0.125) | (0.142) | (0.111) | (0.128) |
| Constant | 0.278*** | 0.277 | 0.000 | -0.020 |
|  | (0.072) | (0.233) | (0.070) | (0.204) |
| Controls | No | Yes | No | Yes |
| R-squared | 0.077 | 0.231 | 0.224 | 0.371 |
| N | 200 | 185 | 182 | 170 |

**Panel B: Indemnity v. Traditional Insurance**

|  | Full Sample | |
| --- | --- | --- |
| Traditional Insurance (Moral Hazard) | 0.207* | 0.172 |
|  | (0.095) | (0.110) |
| Value | 0.235* | 0.274* |
|  | (0.095) | (0.108) |
| Traditional Insurance X Value | -0.053 | -0.074 |
|  | (0.133) | (0.156) |
| Constant | 0.235*** | 0.201 |
|  | (0.067) | (0.307) |
| Controls | No | Yes |
| R-squared | 0.079 | 0.241 |
| N | 210 | 190 |
