# Supplementary material for: Distinguishing moral hazard from access for high-cost healthcare under insurance
Source: PLoS One. 2020 Apr 17;15(4):e0231768. doi: 10.1371/journal.pone.0231768 (PMC7164657; doi:10.1371/journal.pone.0231768)
Supplement: S7 Table — (DOCX) [file pone.0231768.s007.docx]

**Table S7: Cardiovascular: Coronary Artery Disease**

**Panel A: No Insurance v. Indemnity**

|  | Full Sample | | Impossibility Screened | |
| --- | --- | --- | --- | --- |
| Indemnity (Access) | 0.404*** | 0.541*** | 0.591*** | 0.697*** |
|  | (0.102) | (0.120) | (0.093) | (0.111) |
| Value | 0.110 | 0.190 | 0.023 | 0.116 |
|  | (0.093) | (0.109) | (0.093) | (0.118) |
| Indemnity X Value | -0.040 | -0.184 | 0.047 | -0.064 |
|  | (0.140) | (0.164) | (0.130) | (0.162) |
| Constant | 0.326*** | 0.267 | 0.139* | 0.283 |
|  | (0.068) | (0.287) | (0.066) | (0.266) |
| Controls | No | Yes | No | Yes |
| R-squared | 0.152 | 0.354 | 0.383 | 0.545 |
| N | 178 | 157 | 150 | 130 |

**Panel B: Indemnity v. Traditional Insurance**

|  | Full Sample | |
| --- | --- | --- |
| Traditional Insurance (Moral Hazard) | -0.097 | -0.214 |
|  | (0.096) | (0.113) |
| Value | 0.070 | 0.013 |
|  | (0.101) | (0.120) |
| Traditional Insurance X Value | 0.093 | 0.219 |
|  | (0.136) | (0.164) |
| Constant | 0.730*** | 1.233** |
|  | (0.072) | (0.377) |
| Controls | No | Yes |
| R-squared | 0.026 | 0.281 |
| N | 170 | 147 |
