# Supplementary material for: Distinguishing moral hazard from access for high-cost healthcare under insurance
Source: PLoS One. 2020 Apr 17;15(4):e0231768. doi: 10.1371/journal.pone.0231768 (PMC7164657; doi:10.1371/journal.pone.0231768)
Supplement: S8 Table — (DOCX) [file pone.0231768.s008.docx]

**Table S8: Gastrointestinal Disease: GERD**

**Panel A: No Insurance v. Indemnity**

|  | Full Sample | | Impossibility Screened | |
| --- | --- | --- | --- | --- |
| Indemnity (Access) | 0.257** | 0.208 | 0.395*** | 0.363*** |
|  | (0.095) | (0.108) | (0.090) | (0.107) |
| Value | -0.055 | -0.119 | -0.005 | -0.058 |
|  | (0.091) | (0.102) | (0.089) | (0.102) |
| Indemnity X Value | 0.182 | 0.275 | 0.132 | 0.196 |
|  | (0.133) | (0.151) | (0.126) | (0.147) |
| Constant | 0.255*** | 0.438 | 0.116 | 0.363 |
|  | (0.064) | (0.288) | (0.064) | (0.281) |
| Controls | No | Yes | No | Yes |
| R-squared | 0.138 | 0.308 | 0.246 | 0.373 |
| N | 191 | 183 | 178 | 170 |

**Panel B: Indemnity v. Traditional Insurance**

|  | Full Sample | |
| --- | --- | --- |
| Traditional Insurance (Moral Hazard) | -0.029 | -0.076 |
|  | (0.101) | (0.119) |
| Value | 0.127 | 0.089 |
|  | (0.106) | (0.122) |
| Traditional Insurance X Value | -0.119 | -0.099 |
|  | (0.145) | (0.170) |
| Constant | 0.512*** | 0.736 |
|  | (0.076) | (0.447) |
| Controls | No | Yes |
| R-squared | 0.016 | 0.145 |
| N | 193 | 180 |
