# Supplementary material for: Distinguishing moral hazard from access for high-cost healthcare under insurance
Source: PLoS One. 2020 Apr 17;15(4):e0231768. doi: 10.1371/journal.pone.0231768 (PMC7164657; doi:10.1371/journal.pone.0231768)
Supplement: S9 Table — (DOCX) [file pone.0231768.s009.docx]

**Table S9: Age-Related Macular Degeneration (AMD)**

**Panel A: No Insurance v. Indemnity**

|  | Full Sample | | Impossibility Screened | |
| --- | --- | --- | --- | --- |
| Indemnity (Access) | 0.215* | 0.288* | 0.348*** | 0.397*** |
|  | (0.102) | (0.116) | (0.099) | (0.115) |
| Value | 0.275** | 0.341** | 0.212* | 0.259* |
|  | (0.100) | (0.114) | (0.103) | (0.116) |
| Indemnity X Value | 0.080 | 0.021 | 0.142 | 0.065 |
|  | (0.141) | (0.156) | (0.138) | (0.155) |
| Constant | 0.200* | -0.094 | 0.067 | -0.200 |
|  | (0.079) | (0.291) | (0.079) | (0.300) |
| Controls | No | Yes | No | Yes |
| R-squared | 0.134 | 0.331 | 0.235 | 0.408 |
| N | 186 | 169 | 165 | 152 |

**Panel B: Indemnity v. Traditional Insurance**

|  | Full Sample | |
| --- | --- | --- |
| Traditional Insurance (Moral Hazard) | 0.044 | 0.037 |
|  | (0.086) | (0.092) |
| Value | 0.354*** | 0.302** |
|  | (0.096) | (0.108) |
| Traditional Insurance X Value | 0.033 | 0.079 |
|  | (0.129) | (0.145) |
| Constant | 0.415*** | 0.040 |
|  | (0.063) | (0.292) |
| Controls | No | Yes |
| R-squared | 0.149 | 0.320 |
| N | 205 | 189 |
