# Supplementary material for: Distinguishing moral hazard from access for high-cost healthcare under insurance
Source: PLoS One. 2020 Apr 17;15(4):e0231768. doi: 10.1371/journal.pone.0231768 (PMC7164657; doi:10.1371/journal.pone.0231768)
Supplement: S10 Table — (DOCX) [file pone.0231768.s010.docx]

**Table S10: Skin/Autoimmune disease: Psoriasis**

**Panel A: No Insurance v. Indemnity**

|  | Full Sample | | Impossibility Screened | |
| --- | --- | --- | --- | --- |
| Indemnity (Access) | -0.030 | -0.000 | 0.121 | 0.117 |
|  | (0.082) | (0.106) | (0.068) | (0.092) |
| Value | -0.036 | 0.032 | -0.019 | 0.006 |
|  | (0.078) | (0.099) | (0.067) | (0.091) |
| Indemnity X Value | 0.315* | 0.307* | 0.297** | 0.323* |
|  | (0.122) | (0.152) | (0.100) | (0.132) |
| Constant | 0.193*** | 0.562 | 0.042 | 0.105 |
|  | (0.054) | (0.326) | (0.047) | (0.326) |
| Controls | No | Yes | No | Yes |
| R-squared | 0.064 | 0.190 | 0.196 | 0.272 |
| N | 185 | 167 | 169 | 151 |

**Panel B: Indemnity v. Traditional Insurance**

|  | Full Sample | |
| --- | --- | --- |
| Traditional Insurance (Moral Hazard) | 0.176 | 0.104 |
|  | (0.094) | (0.107) |
| Value | 0.278** | 0.310* |
|  | (0.106) | (0.122) |
| Traditional Insurance X Value | -0.254 | -0.206 |
|  | (0.141) | (0.164) |
| Constant | 0.163* | 0.038 |
|  | (0.071) | (0.256) |
| Controls | No | Yes |
| R-squared | 0.043 | 0.237 |
| N | 177 | 160 |
