# Supplementary material for: Distinguishing moral hazard from access for high-cost healthcare under insurance
Source: PLoS One. 2020 Apr 17;15(4):e0231768. doi: 10.1371/journal.pone.0231768 (PMC7164657; doi:10.1371/journal.pone.0231768)
Supplement: S11 Table — (DOCX) [file pone.0231768.s011.docx]

**Table S11: Orthopedic Disease: Degenerative Arthritis**

**Panel A: No Insurance v. Indemnity**

|  | Full Sample | | Impossibility Screened | |
| --- | --- | --- | --- | --- |
| Indemnity (Access) | 0.439*** | 0.480*** | 0.557*** | 0.582*** |
|  | (0.092) | (0.104) | (0.082) | (0.093) |
| Value | -0.008 | 0.022 | -0.053 | -0.026 |
|  | (0.086) | (0.097) | (0.081) | (0.091) |
| Indemnity X Value | -0.015 | -0.037 | 0.030 | 0.013 |
|  | (0.124) | (0.138) | (0.111) | (0.126) |
| Constant | 0.191** | 0.517 | 0.073 | 0.367 |
|  | (0.065) | (0.283) | (0.060) | (0.290) |
| Controls | No | Yes | No | Yes |
| R-squared | 0.194 | 0.285 | 0.364 | 0.446 |
| N | 209 | 193 | 193 | 179 |

**Panel B: Indemnity v. Traditional Insurance**

|  | Full Sample | |
| --- | --- | --- |
| Traditional Insurance (Moral Hazard) | -0.050 | -0.100 |
|  | (0.099) | (0.111) |
| Value | -0.023 | -0.014 |
|  | (0.097) | (0.110) |
| Traditional Insurance X Value | 0.128 | 0.189 |
|  | (0.135) | (0.155) |
| Constant | 0.630*** | 0.892** |
|  | (0.072) | (0.318) |
| Controls | No | Yes |
| R-squared | 0.007 | 0.145 |
| N | 209 | 192 |
