# Supplementary material for: Distinguishing moral hazard from access for high-cost healthcare under insurance
Source: PLoS One. 2020 Apr 17;15(4):e0231768. doi: 10.1371/journal.pone.0231768 (PMC7164657; doi:10.1371/journal.pone.0231768)
Supplement: S12 Table — (DOCX) [file pone.0231768.s012.docx]

**Table S12: Neurologic Disease: Degenerative Lower Spine Disease**

**Panel A: No Insurance v. Indemnity**

|  | Full Sample | | Impossibility Screened | |
| --- | --- | --- | --- | --- |
| Indemnity (Access) | 0.038 | 0.105 | 0.177* | 0.268** |
|  | (0.089) | (0.108) | (0.073) | (0.090) |
| Value | 0.152 | 0.174 | -0.043 | -0.043 |
|  | (0.089) | (0.099) | (0.082) | (0.092) |
| Indemnity X Value | 0.360** | 0.254 | 0.554*** | 0.458*** |
|  | (0.127) | (0.145) | (0.108) | (0.124) |
| Constant | 0.182** | 0.383 | 0.043 | 0.334 |
|  | (0.058) | (0.294) | (0.050) | (0.265) |
| Controls | No | Yes | No | Yes |
| R-squared | 0.218 | 0.356 | 0.448 | 0.556 |
| N | 190 | 175 | 168 | 156 |

**Panel B: Indemnity v. Traditional Insurance**

|  | Full Sample | |
| --- | --- | --- |
| Traditional Insurance (Moral Hazard) | 0.249** | 0.140 |
|  | (0.092) | (0.098) |
| Value | 0.511*** | 0.409*** |
|  | (0.096) | (0.105) |
| Traditional Indemnity X Value | -0.224 | -0.058 |
|  | (0.133) | (0.145) |
| Constant | 0.220** | 0.833*** |
|  | (0.072) | (0.235) |
| Controls | No | Yes |
| R-squared | 0.170 | 0.373 |
| N | 198 | 182 |
