# Supplementary material for: Distinguishing moral hazard from access for high-cost healthcare under insurance
Source: PLoS One. 2020 Apr 17;15(4):e0231768. doi: 10.1371/journal.pone.0231768 (PMC7164657; doi:10.1371/journal.pone.0231768)
Supplement: S1 Data — (DOCX) [file pone.0231768.s013.docx]

Robertson, Yuan, Zhang, & Joiner (2020)

# **Supplemental Information 2 –** **Subject Recruitment and Incentives, Survey Instrument, and Stimulus**

**Note:** Vignette Letters (A-H) correspond to different medical situations (e.g., adenocarcinoma versus metastatic cancer), and high/low refers to different levels of healthcare value (e.g., on-label with randomized trials versus off-label with mere expert opinion).

Contents

[Informed consent 3](#_Toc30229421)

[Demographics Base/Universal 3](#_Toc30229422)

[Adenocarcinoma - Vignette A/High/Indemnity 7](#_Toc30229423)

[Adenocarcinoma - Vignette A/Low/Indemnity 8](#_Toc30229424)

[Adenocarcinoma - Vignette A/Low/Uninsured 10](#_Toc30229425)

[Adenocarcinoma - Vignette A/High/Uninsured 11](#_Toc30229426)

[Adenocarcinoma - Vignette A/Low/Traditional 12](#_Toc30229427)

[Adenocarcinoma - Vignette A/High/Traditional 13](#_Toc30229428)

[Metastatic - VignetteB/High/Indemity 14](#_Toc30229429)

[Metastatic - VignetteB/Low/Indemity 15](#_Toc30229430)

[Metastatic - VignetteB/High/Traditional 17](#_Toc30229431)

[Metastatic - VignetteB/Low/Traditional 18](#_Toc30229432)

[Metastatic - VignetteB/High/Uninsured 19](#_Toc30229433)

[Metastatic - VignetteB/Low/Uninsured 20](#_Toc30229434)

[CAD - VignetteC/High/Indemnity 21](#_Toc30229435)

[CAD - VignetteC/Low/Indemnity 22](#_Toc30229436)

[CAD - VignetteC/High/Traditional 24](#_Toc30229437)

[CAD - VignetteC/Low/Traditional 25](#_Toc30229438)

[CAD - VignetteC/Low/Uninsured 26](#_Toc30229439)

[CAD - VignetteC/High/Uninsured 27](#_Toc30229440)

[GERD - VignetteD/High/Indemnity 28](#_Toc30229441)

[GERD - VignetteD/Low/Indemnity 29](#_Toc30229442)

[GERD - VignetteD/High/Traditional 31](#_Toc30229443)

[GERD - VignetteD/Low/Traditional 32](#_Toc30229444)

[GERD - VignetteD/Low/Uninsured 33](#_Toc30229445)

[GERD - VignetteD/High/Uninsured 34](#_Toc30229446)

[AMD - VignetteE/Low/Uninsured 36](#_Toc30229447)

[AMD - VignetteE/High/Uninsured 37](#_Toc30229448)

[AMD - VignetteE/Low/Traditional 38](#_Toc30229449)

[AMD - VignetteE/High/Traditional 39](#_Toc30229450)

[AMD - VignetteE/Low/Indemnity 40](#_Toc30229451)

[AMD - VignetteE/High/Indemnity 41](#_Toc30229452)

[Psoriasis - VignetteF/High/Indemnity 42](#_Toc30229453)

[Psoriasis - VignetteF/High/Uninsured 45](#_Toc30229454)

[Psoriasis - VignetteF/High/Traditional 47](#_Toc30229455)

[Psoriasis - VignetteF/Low/Indemnity 48](#_Toc30229456)

[ArthKnee - VignetteG/High/Indemnity 49](#_Toc30229457)

[ArthKnee - VignetteG/Low/Indemnity 50](#_Toc30229458)

[ArthKnee - VignetteG/High/Traditional 52](#_Toc30229459)

[ArthKnee - VignetteG/Low/Traditional 53](#_Toc30229460)

[ArthKnee - VignetteG/High/Uninsured 54](#_Toc30229461)

[ArthKnee - VignetteG/Low/Uninsured 55](#_Toc30229462)

[ArthSpine - VignetteH/High/Indemnity 56](#_Toc30229463)

[ArthSpine - VignetteH/High/Traditional 57](#_Toc30229464)

[ArthSpine - VignetteH/High/Uninsured 58](#_Toc30229465)

[ArthSpine - VignetteH/Low/Traditional 59](#_Toc30229466)

[ArthSpine - VignetteH/Low/Uninsured 60](#_Toc30229467)

[ArthSpine - VignetteH/Low/Indemnity 61](#_Toc30229468)

[Closing 63](#_Toc30229469)

# Survey Sampling International Methods

The account representative for Dynata (formerly Survey Sampling International) disclosed the following information about their methods for subject recruitment and incentivization:

For recruiting, Dynata uses invitations of all types including e-mail invitations, phone alerts, banners and messaging on panel community sites to include people with a diversity of motivations to take part in research. At the time of enrollment, new panelists are asked to join an online market/survey research panel. At this point it is made clear that it is not part of a sales process. Our survey invitations provide only basic links and information that is non-leading. Panelists are supported by a dedicated team and have the option to unsubscribe at any time. Our panel management is compliant with market research industry standards, data protection and privacy laws.

Regarding incentives, Dynata offers great diversity in incentives as some people are motivated by cash, points or by being able to donate to charity. Others are motivated by the chance to make a difference, make their voice heard, have fun taking a survey, helping out or having a say in the products and services of the future. Others are motivated by learning opportunities provided by the survey or by the promise of receiving information after taking it. Dynata aims to respond to all of these individual motivations, in order to provide a sample which is diverse and as representative as possible of the target population.

Dynata uses a reasonable level of reward based on the amount of effort required, the population and appropriate regional customs. Regardless of the type of incentive, the value is the same for every respondent in a given study.

Dynata continues to invest in research-on-research into the motivation of online research participants, and continually adjusts its reward offerings based on these findings, as well as current academic thinking about motivation and industry best practices.

# Informed consent

Q14
It will take about 10 minutes to participate in this research.  We will ask you to imagine yourself in a difficult medical situation and then decide what you would do.  **You will have to read several paragraphs, and you will be quizzed on what you read.  So, please only accept this assignment if you are willing and able to be careful.**  

Your participation is voluntary and anonymous.  Please do not submit identifiable information.   Refusal to participate or discontinuation of participation will involve no penalty or loss of benefits to which you are  otherwise entitled.  If you have any questions, contact chris.robertson@law.arizona.edu.  
 
Press the "next" button if you wish to participate.  Close this browser window if you do not consent.

End of Block: Informed consent

# Demographics Base/Universal

| 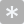 |
| --- |

Q16 What is your year of birth?

________________________________________________________________

Q17 What is the highest level of school you have completed or the highest degree you have received?

- Less than high school degree (1)
- High school graduate (high school diploma or equivalent including GED) (2)
- Some college but no degree (3)
- Associate degree in college (2-year) (4)
- Bachelor's degree in college (4-year) (5)
- Master's degree (6)
- Doctoral degree (7)
- Professional degree (JD, MD) (8)

Q18 Are you of Hispanic, Latino, or Spanish origin?

- No, not of Hispanic, Latino, or Spanish origin? (1)
- Yes, Mexican, Mexican Am., Chicano (2)
- Yes, Puerto Rican (3)
- Yes, Cuban (4)
- Yes, another Hispanic, Latino or Spanish Origin (5)

Q20 Choose one or more races that you consider yourself to be:

- White (1)
- Black, African Am., or negro (2)
- American Indian or Alaska Native (3)
- Asian Indian (4)
- Chinese (5)
- Filipino (6)
- Other Asian (7)
- Japanese (8)
- Korean (9)
- Vietnamese (10)
- Native Hawaiian (11)
- Guamanian or Charmorro (12)
- Samoan (13)
- Other Pacific Islander (14)
- Some other race. (15)

Q21 What is your sex?

- Male (1)
- Female (2)

Q22 Information about income is very important to understand.  Would you please give your best estimate?Please indicate the answer that includes your entire household income in (previous year) before taxes.

- Less than $10,000 (1)
- $10,000 to $19,999 (2)
- $20,000 to $29,999 (3)
- $30,000 to $39,999 (4)
- $40,000 to $49,999 (5)
- $50,000 to $59,999 (6)
- $60,000 to $69,999 (7)
- $70,000 to $79,999 (8)
- $80,000 to $89,999 (9)
- $90,000 to $99,999 (10)
- $100,000 to $149,999 (11)
- $150,000 or more (12)

| 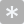 |
| --- |

Q36 Purchasing power is different.  If you needed to make a very important purchase, what is the **maximum** amount of money that you could access to make that purchase within one month from today?      

Please consider all sources of money or credit accessible to you, including cash, cars that you could sell or refinance, consumer credit, home equity that you could access through a loan, personal loans from friends or family, money in health savings accounts, money in retirement accounts, gifts, and donations.

Please enter number of dollars that is the MAXIMUM amount that you could spend if absolutely necessary to make such a purchase, if you absolutely had to do so.  We understand that this may not be exact; just your best estimate please.  (Omit the dollar sign.)

________________________________________________________________

Q37 Please explain briefly how you would come up with that amount of money.  From what sources would you draw to reach that amount?  What might you have to give up?

________________________________________________________________

________________________________________________________________

________________________________________________________________

________________________________________________________________

________________________________________________________________

Q230 On the next screen, you will read a story.  Please imagine yourself in the story.  Then you will be asked to make a decision about what you would do in that story.

End of Block: Demographics Base/Universal

# Adenocarcinoma - Vignette A/High/Indemnity

Q69 You are generally in good health, but over the last month, blood has occasionally appeared in your stool, when you used the bathroom. You make an appointment to see a doctor. After receiving normal results from some tests, the doctor suggests a colonoscopy to determine the cause of the bleeding. The test reveals a mass, and so a biopsy is performed.   The biopsy confirms that the bleeding is caused by adenocarcinoma of the colon, the most common type of colon cancer. Your oncologist performs further tests to determine if the cancer has spread beyond the colon, since this will determine the type of treatment you will get and for how long. The tests, including a CT scan, are negative, indicating that the cancer is limited to the colon. Therefore, surgery is scheduled to remove the tumor, and hopefully provide a complete cure.   At surgery, the surgeons discover that the tumor has spread past the wall of the colon and infected two small lymph nodes. These are the first sites outside of the colon where cancer usually spreads. These lymph nodes were too small to be detected by the CT scan or the other tests done before the surgery. These findings substantially change the prognosis. It is unlikely that surgery alone will result in a full recovery, since it is almost a certainty that the tumor has spread to other sites that are still too small to be detected.   Hence, the oncologist tells you that chemotherapy is the standard treatment. The oncologist estimates that the chance of survival from this cancer, with the standard chemotherapy treatment, is 70%. The chemotherapy will last for 6 months. Common side effects include hair loss, mouth sores, nausea and vomiting, diarrhea, fatigue, and increased chance of infections, from having too few white blood cells.   All of this information is overwhelming, and the doctor leaves the room to allow you time to think about what you have just learned.

Q129 Please refer to the story specifically, and share a sentence describing how you would feel in this medical situation, and your thoughts about your treatment options described so far.

________________________________________________________________

| Page Break |  |
| --- | --- |

Q130 Upon returning, the oncologist suggests that another drug, Bucarin, could be added to the standard chemotherapy regimen to improve the chances for a cure. Bucarin has a different way of acting than standard chemotherapy does, which is the reason your oncologist is suggesting it could be added. The potential side effects are also different, including excessive and potentially fatal bleeding. 
The FDA has approved Bucarin for colon cancer. Two large randomized trials have shown that it improves the chances of survival.  In this case, Bucarin will cost an additional $80,000. Please imagine you have full health insurance, and you have already met your annual deductible. Your insurance company has a unique program, in which they cover special drugs with a cash payment, called an indemnity (similar to car insurance). Because your physician has indicated that you are a candidate for Bucarin, **you receive a $80,000 cash payment from your insurance company. You receive the money regardless of whether you decide to take the Bucarin**. You decide whether you spend the cash on the Bucarin or instead keep the money for whatever other purposes you may choose. In deciding, please be realistic.

Q280 On the next page you will share your decision.  First, now please write two sentences putting in your own words, (1) your understanding of the treatment options, and (2) your insurance situation, as described above.

________________________________________________________________

End of Block: Adenocarcinoma - Vignette A/High/Indemnity

# Adenocarcinoma - Vignette A/Low/Indemnity

Q81   You are generally in good health, but over the last month, blood has occasionally appeared in your stool, when you used the bathroom. You make an appointment to see a doctor. After receiving normal results from some tests, the doctor suggests a colonoscopy to determine the cause of the bleeding. The test reveals a mass, and so a biopsy is performed.   The biopsy confirms that the bleeding is caused by adenocarcinoma of the colon, the most common type of colon cancer. Your oncologist performs further tests to determine if the cancer has spread beyond the colon, since this will determine the type of treatment you will get and for how long. The tests, including a CT scan, are negative, indicating that the cancer is limited to the colon. Therefore, surgery is scheduled to remove the tumor, and hopefully provide a complete cure.   At surgery, the surgeons discover that the tumor has spread past the wall of the colon and infected two small lymph nodes. These are the first sites outside of the colon where cancer usually spreads. These lymph nodes were too small to be detected by the CT scan or the other tests done before the surgery. These findings substantially change the prognosis. It is unlikely that surgery alone will result in a full recovery, since it is almost a certainty that the tumor has spread to other sites that are still too small to be detected.   Hence, the oncologist tells you that chemotherapy is the standard treatment. The oncologist estimates that the chance of survival from this cancer, with the standard chemotherapy treatment, is 70%. The chemotherapy will last for 6 months. Common side effects include hair loss, mouth sores, nausea and vomiting, diarrhea, fatigue, and increased chance of infections, from having too few white blood cells.   All of this information is overwhelming, and the doctor leaves the room to allow you time to think about what you have just learned.

Q132 Please refer to the story specifically, and share a sentence describing how you would feel in this medical situation, and your thoughts about your treatment options described so far.

________________________________________________________________

| Page Break |  |
| --- | --- |

Q133 Upon returning, the oncologist suggests that another drug, Bucarin, could be added to the standard chemotherapy regimen to improve the chances for a cure. Bucarin has a different way of acting than standard chemotherapy does, which is the reason your oncologist is suggesting it could be added. The potential side effects are also different, including excessive and potentially fatal bleeding.

Your oncologist says that the FDA has approved Bucarin for use in other cancers, but not for use in colon cancer, because large rigorous studies have not been done for colon cancer. Because he has personally had good experience with the drug, your oncologist recommends “off-label” use of Bucarin despite this limitation.    In this case, Bucarin will cost an additional $80,000. Please imagine you have full health insurance, and you have already met your annual deductible. Your insurance company has a unique program, in which they cover special drugs with a cash payment, called an indemnity (similar to car insurance). Because your physician has indicated that you are a candidate for Bucarin, **you receive a $80,000 cash payment from your insurance company. You receive the money regardless of whether you decide to take the Bucarin**. You decide whether you spend the cash on the Bucarin or instead keep the money for whatever other purposes you may choose. In deciding, please be realistic.

Q279 On the next page you will share your decision.  First, now please write two sentences putting in your own words, (1) your understanding of the treatment options, and (2) your insurance situation, as described above.

________________________________________________________________

End of Block: Adenocarcinoma - Vignette A/Low/Indemnity

# Adenocarcinoma - Vignette A/Low/Uninsured

Q78 You are generally in good health, but over the last month, blood has occasionally appeared in your stool, when you used the bathroom. You make an appointment to see a doctor. After receiving normal results from some tests, the doctor suggests a colonoscopy to determine the cause of the bleeding. The test reveals a mass, and so a biopsy is performed.   The biopsy confirms that the bleeding is caused by adenocarcinoma of the colon, the most common type of colon cancer. Your oncologist performs further tests to determine if the cancer has spread beyond the colon, since this will determine the type of treatment you will get and for how long. The tests, including a CT scan, are negative, indicating that the cancer is limited to the colon. Therefore, surgery is scheduled to remove the tumor, and hopefully provide a complete cure.   At surgery, the surgeons discover that the tumor has spread past the wall of the colon and infected two small lymph nodes. These are the first sites outside of the colon where cancer usually spreads. These lymph nodes were too small to be detected by the CT scan or the other tests done before the surgery. These findings substantially change the prognosis. It is unlikely that surgery alone will result in a full recovery, since it is almost a certainty that the tumor has spread to other sites that are still too small to be detected.   Hence, the oncologist tells you that chemotherapy is the standard treatment. The oncologist estimates that the chance of survival from this cancer, with the standard chemotherapy treatment, is 70%. The chemotherapy will last for 6 months. Common side effects include hair loss, mouth sores, nausea and vomiting, diarrhea, fatigue, and increased chance of infections, from having too few white blood cells.   All of this information is overwhelming, and the doctor leaves the room to allow you time to think about what you have just learned.

Q134 Please refer to the story specifically, and share a sentence describing how you would feel in this medical situation, and your thoughts about your treatment options described so far.

________________________________________________________________

| Page Break |  |
| --- | --- |

Q135 Upon returning, the oncologist suggests that another drug, Bucarin, could be added to the standard chemotherapy regimen to improve the chances for a cure. Bucarin has a different way of acting than standard chemotherapy does, which is the reason your oncologist is suggesting it could be added. The potential side effects are also different, including excessive and potentially fatal bleeding.  

 Your oncologist says that the FDA has approved Bucarin for use in other cancers, but not for use in colon cancer, because large rigorous studies have not been done for colon cancer. Because he has personally had good experience with the drug, your oncologist recommends “off-label” use of Bucarin despite this limitation.   

In this case, Bucarin will cost an additional $80,000. Please imagine that **you currently do not have health insurance**, however you do have the same amount of wealth and credit that you actually have in the real world (the amount you provided in your answer above). So, only if you have enough money available, you can pay for the Bucarin yourself. Think back to your answer to the purchasing power above. If you do have that much purchasing power, please consider however, other things you may prefer to do with the money. Please be realistic.

Q278 On the next page you will share your decision.  First, now please write two sentences putting in your own words, (1) your understanding of the treatment options, and (2) your insurance situation, as described above.

________________________________________________________________

End of Block: Adenocarcinoma - Vignette A/Low/Uninsured

# Adenocarcinoma - Vignette A/High/Uninsured

Q72 You are generally in good health, but over the last month, blood has occasionally appeared in your stool, when you used the bathroom. You make an appointment to see a doctor. After receiving normal results from some tests, the doctor suggests a colonoscopy to determine the cause of the bleeding. The test reveals a mass, and so a biopsy is performed.   The biopsy confirms that the bleeding is caused by adenocarcinoma of the colon, the most common type of colon cancer. Your oncologist performs further tests to determine if the cancer has spread beyond the colon, since this will determine the type of treatment you will get and for how long. The tests, including a CT scan, are negative, indicating that the cancer is limited to the colon. Therefore, surgery is scheduled to remove the tumor, and hopefully provide a complete cure.   At surgery, the surgeons discover that the tumor has spread past the wall of the colon and infected two small lymph nodes. These are the first sites outside of the colon where cancer usually spreads. These lymph nodes were too small to be detected by the CT scan or the other tests done before the surgery. These findings substantially change the prognosis. It is unlikely that surgery alone will result in a full recovery, since it is almost a certainty that the tumor has spread to other sites that are still too small to be detected.   Hence, the oncologist tells you that chemotherapy is the standard treatment. The oncologist estimates that the chance of survival from this cancer, with the standard chemotherapy treatment, is 70%. The chemotherapy will last for 6 months. Common side effects include hair loss, mouth sores, nausea and vomiting, diarrhea, fatigue, and increased chance of infections, from having too few white blood cells.   All of this information is overwhelming, and the doctor leaves the room to allow you time to think about what you have just learned.

Q136 Please refer to the story specifically, and share a sentence describing how you would feel in this medical situation, and your thoughts about your treatment options described so far.

________________________________________________________________

| Page Break |  |
| --- | --- |

Q137   Upon returning, the oncologist suggests that another drug, Bucarin, could be added to the standard chemotherapy regimen to improve the chances for a cure. Bucarin has a different way of acting than standard chemotherapy does, which is the reason your oncologist is suggesting it could be added. The potential side effects are also different, including excessive and potentially fatal bleeding.

The FDA has approved Bucarin for colon cancer. Two large randomized trials have shown that it improves the chances of survival. 
  
In this case, Bucarin will cost an additional $80,000. Please imagine that **you currently do not have health insurance**, however you do have the same amount of wealth and credit that you actually have in the real world (the amount you provided in your answer above). So, only if you have enough money available, you can pay for the Bucarin yourself. Think back to your answer to the purchasing power above. If you do have that much purchasing power, please consider however, other things you may prefer to do with the money. Please be realistic.

Q277 On the next page you will share your decision.  First, now please write two sentences putting in your own words, (1) your understanding of the treatment options, and (2) your insurance situation, as described above.

________________________________________________________________

End of Block: Adenocarcinoma - Vignette A/High/Uninsured

# Adenocarcinoma - Vignette A/Low/Traditional

Q75 You are generally in good health, but over the last month, blood has occasionally appeared in your stool, when you used the bathroom. You make an appointment to see a doctor. After receiving normal results from some tests, the doctor suggests a colonoscopy to determine the cause of the bleeding. The test reveals a mass, and so a biopsy is performed.

The biopsy confirms that the bleeding is caused by adenocarcinoma of the colon, the most common type of colon cancer. Your oncologist performs further tests to determine if the cancer has spread beyond the colon, since this will determine the type of treatment you will get and for how long. The tests, including a CT scan, are negative, indicating that the cancer is limited to the colon. Therefore, surgery is scheduled to remove the tumor, and hopefully provide a complete cure.   At surgery, the surgeons discover that the tumor has spread past the wall of the colon and infected two small lymph nodes. These are the first sites outside of the colon where cancer usually spreads. These lymph nodes were too small to be detected by the CT scan or the other tests done before the surgery. These findings substantially change the prognosis. It is unlikely that surgery alone will result in a full recovery, since it is almost a certainty that the tumor has spread to other sites that are still too small to be detected.

Hence, the oncologist tells you that chemotherapy is the standard treatment. The oncologist estimates that the chance of survival from this cancer, with the standard chemotherapy treatment, is 70%. The chemotherapy will last for 6 months. Common side effects include hair loss, mouth sores, nausea and vomiting, diarrhea, fatigue, and increased chance of infections, from having too few white blood cells.   All of this information is overwhelming, and the doctor leaves the room to allow you time to think about what you have just learned.

Q138 Please refer to the story specifically, and share a sentence describing how you would feel in this medical situation, and your thoughts about your treatment options described so far.

________________________________________________________________

| Page Break |  |
| --- | --- |

Q139 Upon returning, the oncologist suggests that another drug, Bucarin, could be added to the standard chemotherapy regimen to improve the chances for a cure. Bucarin has a different way of acting than standard chemotherapy does, which is the reason your oncologist is suggesting it could be added. The potential side effects are also different, including excessive and potentially fatal bleeding.

 Your oncologist says that the FDA has approved Bucarin for use in other cancers, but not for use in colon cancer, because large rigorous studies have not been done for colon cancer. Because he has personally had good experience with the drug, your oncologist recommends “off-label” use of Bucarin despite this limitation.     
 In this case, Bucarin will cost an additional $80,000. Please imagine **you have full health insurance**, and you have already met your annual deductible, so there is no additional cost to you out of pocket, if you choose to take the Bucarin treatment. In deciding, please be realistic.

Q276 On the next page you will share your decision.  First, now please write two sentences putting in your own words, (1) your understanding of the treatment options, and (2) your insurance situation, as described above.

________________________________________________________________

End of Block: Adenocarcinoma - Vignette A/Low/Traditional

# Adenocarcinoma - Vignette A/High/Traditional

Q46 You are generally in good health, but over the last month, blood has occasionally appeared in your stool, when you used the bathroom. You make an appointment to see a doctor. After receiving normal results from some tests, the doctor suggests a colonoscopy to determine the cause of the bleeding. The test reveals a mass, and so a biopsy is performed.

The biopsy confirms that the bleeding is caused by adenocarcinoma of the colon, the most common type of colon cancer. Your oncologist performs further tests to determine if the cancer has spread beyond the colon, since this will determine the type of treatment you will get and for how long. The tests, including a CT scan, are negative, indicating that the cancer is limited to the colon. Therefore, surgery is scheduled to remove the tumor, and hopefully provide a complete cure.

At surgery, the surgeons discover that the tumor has spread past the wall of the colon and infected two small lymph nodes. These are the first sites outside of the colon where cancer usually spreads. These lymph nodes were too small to be detected by the CT scan or the other tests done before the surgery. These findings substantially change the prognosis. It is unlikely that surgery alone will result in a full recovery, since it is almost a certainty that the tumor has spread to other sites that are still too small to be detected.

Hence, the oncologist tells you that chemotherapy is the standard treatment. The oncologist estimates that the chance of survival from this cancer, with the standard chemotherapy treatment, is 70%. The chemotherapy will last for 6 months. Common side effects include hair loss, mouth sores, nausea and vomiting, diarrhea, fatigue, and increased chance of infections, from having too few white blood cells.   All of this information is overwhelming, and the doctor leaves the room to allow you time to think about what you have just learned.

Q140 Please refer to the story specifically, and share a sentence describing how you would feel in this medical situation, and your thoughts about your treatment options described so far.

________________________________________________________________

| Page Break |  |
| --- | --- |

Q141 Upon returning, the oncologist suggests that another drug, Bucarin, could be added to the standard chemotherapy regimen to improve the chances for a cure. Bucarin has a different way of acting than standard chemotherapy does, which is the reason your oncologist is suggesting it could be added. The potential side effects are also different, including excessive and potentially fatal bleeding.

The FDA has approved Bucarin for colon cancer. Two large randomized trials have shown that it improves the chances of survival.

In this case, Bucarin will cost an additional $80,000. Please imagine **you have full health insurance**, and you have already met your annual deductible, so there is no additional cost to you out of pocket, if you choose to take the Bucarin treatment. In deciding, please be realistic.

Q275 On the next page you will share your decision.  First, now please write two sentences putting in your own words, (1) your understanding of the treatment options, and (2) your insurance situation, as described above.

________________________________________________________________

End of Block: Adenocarcinoma - Vignette A/High/Traditional

# Metastatic - VignetteB/High/Indemity

Q48 One year ago, you were diagnosed with lung cancer. At the time, the cancer had already spread to your liver and your bones, but you were not having any symptoms from the cancer in these locations. You received 6 months of chemotherapy and radiation therapy, which helped a little. Some of the side effects, such as nausea, vomiting and intense fatigue, were disabling. The time and travel involved to get to all of the treatment appointments was inconvenient at best and quite disruptive at times. You were not able to travel or do any of the other things on your bucket list.

Unfortunately, your cancer is now progressing rapidly. The cancer in your liver is worse, and you are having pain from cancer that has spread to your ribs. A scan of your brain now shows a small area where cancer has spread, although you do not yet have symptoms from the cancer in your brain.

When you press for details, your oncologist reluctantly admits that your life expectancy is probably about 6 months with no further therapy, but admits it is hard to say for sure. Your first course of chemotherapy and radiation therapy was very disabling, and you are not sure you want to do it again.

Q142 Please refer to the story specifically, and share a sentence describing how you would feel in this medical situation, and your thoughts about your treatment options described so far.

________________________________________________________________

| Page Break |  |
| --- | --- |

Q143 Your oncologist tells you that you may benefit from a newly approved drug (Doloxan) to treat your type of lung cancer. Clinical studies showed that Doloxan increased average survival by 8 months in patients like you who have received standard therapy and are now progressing.

Side effects for people who took Doloxan are quite general, and include loss of appetite, nausea, shortness of breath and constipation. They are usually milder than the side effects you have with standard chemotherapy. Many of these side effects may not even be related to the drug; they are common for people with advanced cancer.

The oncologist says that, instead of getting more treatment with Doloxan, you should think about hospice care. For patients with a different type of lung cancer, one study showed that quality of life is better and even life expectancy was longer with hospice care instead of continuing cancer treatment. The oncologist is not sure if the results apply to your cancer, especially since the new drug treatment with Doloxan is now available, but says that because you are not expected to live more than 6 months, you qualify for home hospice care. With home hospice care, you would still live at home. Your symptoms (for example the bone pain) would be treated, but you would not get any more chemotherapy or radiation. You would have more choice about how you spend your remaining time.  

In this case, Doloxan will cost an additional $125,000. Please imagine you have full health insurance, and you have already met your annual deductible. Your insurance company has a unique program, in which they cover special drugs with a cash payment, called an indemnity (similar to car insurance). Because your physician has indicated that you are a candidate for Doloxan, **you receive a $125,000 cash payment from your insurance company. You receive the money regardless of whether you decide to take the Doloxan**. You decide whether you spend the cash on the Doloxan or instead keep the money for whatever other purposes you may choose. In deciding, please be realistic.

Q274 On the next page you will share your decision.  First, now please write two sentences putting in your own words, (1) your understanding of the treatment options, and (2) your insurance situation, as described above.

________________________________________________________________

End of Block: Metastatic - VignetteB/High/Indemity

# Metastatic - VignetteB/Low/Indemity

Q84
 One year ago, you were diagnosed with lung cancer. At the time, the cancer had already spread to your liver and your bones, but you were not having any symptoms from the cancer in these locations. You received 6 months of chemotherapy and radiation therapy, which helped a little. Some of the side effects, such as nausea, vomiting and intense fatigue, were disabling. The time and travel involved to get to all of the treatment appointments was inconvenient at best and quite disruptive at times. You were not able to travel or do any of the other things on your bucket list.

Unfortunately, your cancer is now progressing rapidly. The cancer in your liver is worse, and you are having pain from cancer that has spread to your ribs. A scan of your brain now shows a small area where cancer has spread, although you do not yet have symptoms from the cancer in your brain.   When you press for details, your oncologist reluctantly admits that your life expectancy is probably about 6 months with no further therapy, but admits it is hard to say for sure. Your first course of chemotherapy and radiation therapy was very disabling, and you are not sure you want to do it again.

Q144 Please refer to the story specifically, and share a sentence describing how you would feel in this medical situation, and your thoughts about your treatment options described so far.

________________________________________________________________

| Page Break |  |
| --- | --- |

Q145 Your oncologist tells you that you may benefit from a newly approved drug (Doloxan) to treat your type of lung cancer. Clinical studies showed that Doloxan caused tumors to stop growing in any way that could be measured for four months. However, these studies did not show any overall survival benefit: patients who took the drug did not live longer than patients who did not take the drug.

  Side effects for people who took Doloxan are quite general, and include loss of appetite, nausea, shortness of breath and constipation. They are usually milder than the side effects you have with standard chemotherapy. Many of these side effects may not even be related to the drug; they are common for people with advanced cancer.

  The oncologist says that, instead of getting more treatment with Doloxan, you should think about hospice care. For patients with a different type of lung cancer, one study showed that quality of life is better and even life expectancy was longer with hospice care instead of continuing cancer treatment. The oncologist is not sure if the results apply to your cancer, especially since the new drug treatment with Doloxan is now available, but says that because you are not expected to live more than 6 months, you qualify for home hospice care. With home hospice care, you would still live at home. Your symptoms (for example the bone pain) would be treated, but you would not get any more chemotherapy or radiation. You would have more choice about how you spend your remaining time.

  In this case, Doloxan will cost an additional $125,000. Please imagine you have full health insurance, and you have already met your annual deductible. Your insurance company has a unique program, in which they cover special drugs with a cash payment, called an indemnity (similar to car insurance). Because your physician has indicated that you are a candidate for Doloxan, **you receive a $125,000 cash payment from your insurance company. You receive the money regardless of whether you decide to take the Doloxan**. You decide whether you spend the cash on the Doloxan or instead keep the money for whatever other purposes you may choose. In deciding, please be realistic.

Q273 On the next page you will share your decision.  First, now please write two sentences putting in your own words, (1) your understanding of the treatment options, and (2) your insurance situation, as described above.

________________________________________________________________

End of Block: Metastatic - VignetteB/Low/Indemity

# Metastatic - VignetteB/High/Traditional

Q87
 One year ago, you were diagnosed with lung cancer. At the time, the cancer had already spread to your liver and your bones, but you were not having any symptoms from the cancer in these locations. You received 6 months of chemotherapy and radiation therapy, which helped a little. Some of the side effects, such as nausea, vomiting and intense fatigue, were disabling. The time and travel involved to get to all of the treatment appointments was inconvenient at best and quite disruptive at times. You were not able to travel or do any of the other things on your bucket list.

  Unfortunately, your cancer is now progressing rapidly. The cancer in your liver is worse, and you are having pain from cancer that has spread to your ribs. A scan of your brain now shows a small area where cancer has spread, although you do not yet have symptoms from the cancer in your brain.   When you press for details, your oncologist reluctantly admits that your life expectancy is probably about 6 months with no further therapy, but admits it is hard to say for sure. Your first course of chemotherapy and radiation therapy was very disabling, and you are not sure you want to do it again.

Q146 Please refer to the story specifically, and share a sentence describing how you would feel in this medical situation, and your thoughts about your treatment options described so far.

________________________________________________________________

| Page Break |  |
| --- | --- |

Q147 Your oncologist tells you that you may benefit from a newly approved drug (Doloxan) to treat your type of lung cancer. Clinical studies showed that Doloxan increased average survival by 8 months in patients like you who have received standard therapy and are now progressing. Side effects for people who took Doloxan are quite general, and include loss of appetite, nausea, shortness of breath and constipation. They are usually milder than the side effects you have with standard chemotherapy. Many of these side effects may not even be related to the drug; they are common for people with advanced cancer. The oncologist says that, instead of getting more treatment with Doloxan, you should think about hospice care. For patients with a different type of lung cancer, one study showed that quality of life is better and even life expectancy was longer with hospice care instead of continuing cancer treatment. The oncologist is not sure if the results apply to your cancer, especially since the new drug treatment with Doloxan is now available, but says that because you are not expected to live more than 6 months, you qualify for home hospice care. With home hospice care, you would still live at home. Your symptoms (for example the bone pain) would be treated, but you would not get any more chemotherapy or radiation. You would have more choice about how you spend your remaining time. 
In this case, Doloxan will cost an additional $125,000. Please imagine **you have full health insurance**, and you have already met your annual deductible, so there is no additional cost to you out of pocket, if you choose to take the Doloxan treatment. In deciding, please be realistic.

Q272 On the next page you will share your decision.  First, now please write two sentences putting in your own words, (1) your understanding of the treatment options, and (2) your insurance situation, as described above.

________________________________________________________________

End of Block: Metastatic - VignetteB/High/Traditional

# Metastatic - VignetteB/Low/Traditional

Q90
 One year ago, you were diagnosed with lung cancer. At the time, the cancer had already spread to your liver and your bones, but you were not having any symptoms from the cancer in these locations. You received 6 months of chemotherapy and radiation therapy, which helped a little. Some of the side effects, such as nausea, vomiting and intense fatigue, were disabling. The time and travel involved to get to all of the treatment appointments was inconvenient at best and quite disruptive at times. You were not able to travel or do any of the other things on your bucket list.   Unfortunately, your cancer is now progressing rapidly. The cancer in your liver is worse, and you are having pain from cancer that has spread to your ribs. A scan of your brain now shows a small area where cancer has spread, although you do not yet have symptoms from the cancer in your brain.   When you press for details, your oncologist reluctantly admits that your life expectancy is probably about 6 months with no further therapy, but admits it is hard to say for sure. Your first course of chemotherapy and radiation therapy was very disabling, and you are not sure you want to do it again.

Q148 Please refer to the story specifically, and share a sentence describing how you would feel in this medical situation, and your thoughts about your treatment options described so far.

________________________________________________________________

| Page Break |  |
| --- | --- |

Q149 Your oncologist tells you that you may benefit from a newly approved drug (Doloxan) to treat your type of lung cancer. Clinical studies showed that Doloxan caused tumors to stop growing in any way that could be measured for four months. However, these studies did not show any overall survival benefit: patients who took the drug did not live longer than patients who did not take the drug.   Side effects for people who took Doloxan are quite general, and include loss of appetite, nausea, shortness of breath and constipation. They are usually milder than the side effects you have with standard chemotherapy. Many of these side effects may not even be related to the drug; they are common for people with advanced cancer.   The oncologist says that, instead of getting more treatment with Doloxan, you should think about hospice care. For patients with a different type of lung cancer, one study showed that quality of life is better and even life expectancy was longer with hospice care instead of continuing cancer treatment. The oncologist is not sure if the results apply to your cancer, especially since the new drug treatment with Doloxan is now available, but says that because you are not expected to live more than 6 months, you qualify for home hospice care. With home hospice care, you would still live at home. Your symptoms (for example the bone pain) would be treated, but you would not get any more chemotherapy or radiation. You would have more choice about how you spend your remaining time.   In this case, Doloxan will cost an additional $125,000. Please imagine **you have full health insurance**, and you have already met your annual deductible, so there is no additional cost to you out of pocket, if you choose to take the Doloxan treatment. In deciding, please be realistic.

Q271 On the next page you will share your decision.  First, now please write two sentences putting in your own words, (1) your understanding of the treatment options, and (2) your insurance situation, as described above.

________________________________________________________________

End of Block: Metastatic - VignetteB/Low/Traditional

# Metastatic - VignetteB/High/Uninsured

Q93 One year ago, you were diagnosed with lung cancer. At the time, the cancer had already spread to your liver and your bones, but you were not having any symptoms from the cancer in these locations. You received 6 months of chemotherapy and radiation therapy, which helped a little. Some of the side effects, such as nausea, vomiting and intense fatigue, were disabling. The time and travel involved to get to all of the treatment appointments was inconvenient at best and quite disruptive at times. You were not able to travel or do any of the other things on your bucket list.   Unfortunately, your cancer is now progressing rapidly. The cancer in your liver is worse, and you are having pain from cancer that has spread to your ribs. A scan of your brain now shows a small area where cancer has spread, although you do not yet have symptoms from the cancer in your brain.   When you press for details, your oncologist reluctantly admits that your life expectancy is probably about 6 months with no further therapy, but admits it is hard to say for sure. Your first course of chemotherapy and radiation therapy was very disabling, and you are not sure you want to do it again.

Q150 Please refer to the story specifically, and share a sentence describing how you would feel in this medical situation, and your thoughts about your treatment options described so far.

________________________________________________________________

| Page Break |  |
| --- | --- |

Q151 Your oncologist tells you that you may benefit from a newly approved drug (Doloxan) to treat your type of lung cancer. Clinical studies showed that Doloxan increased average survival by
 8 months in patients like you who have received standard therapy and are now progressing.   Side effects for people who took Doloxan are quite general, and include loss of appetite, nausea, shortness of breath and constipation. They are usually milder than the side effects you have with standard chemotherapy. Many of these side effects may not even be related to the drug; they are common for people with advanced cancer.   The oncologist says that, instead of getting more treatment with Doloxan, you should think about hospice care. For patients with a different type of lung cancer, one study showed that quality of life is better and even life expectancy was longer with hospice care instead of continuing cancer treatment. The oncologist is not sure if the results apply to your cancer, especially since the new drug treatment with Doloxan is now available, but says that because you are not expected to live more than 6 months, you qualify for home hospice care. With home hospice care, you would still live at home. Your symptoms (for example the bone pain) would be treated, but you would not get any more chemotherapy or radiation. You would have more choice about how you spend your remaining time.   In this case, Doloxan will cost an additional $125,000. Please imagine that **you currently do not have health insurance**, however you do have the same amount of wealth and credit that you actually have in the real world (the amount you provided in your answer above). So, only if you have enough money available, you can pay for the Doloxan yourself. Think back to your answer to the purchasing power above. If you do have that much purchasing power, please consider however, other things you may prefer to do with the money. Please be realistic.

Q270 On the next page you will share your decision.  First, now please write two sentences putting in your own words, (1) your understanding of the treatment options, and (2) your insurance situation, as described above.

________________________________________________________________

End of Block: Metastatic - VignetteB/High/Uninsured

# Metastatic - VignetteB/Low/Uninsured

Q96
 One year ago, you were diagnosed with lung cancer. At the time, the cancer had already spread to your liver and your bones, but you were not having any symptoms from the cancer in these locations. You received 6 months of chemotherapy and radiation therapy, which helped a little. Some of the side effects, such as nausea, vomiting and intense fatigue, were disabling. The time and travel involved to get to all of the treatment appointments was inconvenient at best and quite disruptive at times. You were not able to travel or do any of the other things on your bucket list.   Unfortunately, your cancer is now progressing rapidly. The cancer in your liver is worse, and you are having pain from cancer that has spread to your ribs. A scan of your brain now shows a small area where cancer has spread, although you do not yet have symptoms from the cancer in your brain.   When you press for details, your oncologist reluctantly admits that your life expectancy is probably about 6 months with no further therapy, but admits it is hard to say for sure. Your first course of chemotherapy and radiation therapy was very disabling, and you are not sure you want to do it again.

Q152 Please refer to the story specifically, and share a sentence describing how you would feel in this medical situation, and your thoughts about your treatment options described so far.

________________________________________________________________

| Page Break |  |
| --- | --- |

Q153 Your oncologist tells you that you may benefit from a newly approved drug (Doloxan) to treat your type of lung cancer. Clinical studies showed that Doloxan caused tumors to stop growing in any way that could be measured for four months. However, these studies did not show any overall survival benefit: patients who took the drug did not live longer than patients who did not take the drug.   Side effects for people who took Doloxan are quite general, and include loss of appetite, nausea, shortness of breath and constipation. They are usually milder than the side effects you have with standard chemotherapy. Many of these side effects may not even be related to the drug; they are common for people with advanced cancer.   The oncologist says that, instead of getting more treatment with Doloxan, you should think about hospice care. For patients with a different type of lung cancer, one study showed that quality of life is better and even life expectancy was longer with hospice care instead of continuing cancer treatment. The oncologist is not sure if the results apply to your cancer, especially since the new drug treatment with Doloxan is now available, but says that because you are not expected to live more than 6 months, you qualify for home hospice care. With home hospice care, you would still live at home. Your symptoms (for example the bone pain) would be treated, but you would not get any more chemotherapy or radiation. You would have more choice about how you spend your remaining time.   In this case, Doloxan will cost an additional $125,000. Please imagine that **you currently do not have health insurance**, however you do have the same amount of wealth and credit that you actually have in the real world (the amount you provided in your answer above). So, only if you have enough money available, you can pay for the Doloxan yourself. Think back to your answer to the purchasing power above. If you do have that much purchasing power, please consider however, other things you may prefer to do with the money. Please be realistic.

Q269 On the next page you will share your decision.  First, now please write two sentences putting in your own words, (1) your understanding of the treatment options, and (2) your insurance situation, as described above.

________________________________________________________________

End of Block: Metastatic - VignetteB/Low/Uninsured

# CAD - VignetteC/High/Indemnity

Q50

 Over the last month, you have noticed an occasional feeling of discomfort in your chest, which generally lasts for 5-10 minutes. At first, you thought it was just indigestion, but now you notice that the feeling occurs when you climb stairs and has no relationship to food.   Concerned, you visit your primary care doctor, who does an examination, some blood tests, and an electrocardiogram (EKG). The examination shows a mildly elevated blood pressure, and the blood tests show a moderately elevated cholesterol level. The EKG is normal.   Still, concerned that your symptoms suggested the possibility of heart disease, your doctor refers you to a cardiologist (heart specialist), who recommends taking a stress echocardiogram, which means exercising on a treadmill while your heart is monitored. While exercising, the discomfort in your chest returns, and the EKG shows abnormalities in your heart function. These results strongly suggest that you have a blockage in your coronary arteries. This is confirmed with another test, a coronary angiogram, which shows one area with a lot of blockage.

Q154 Please refer to the story specifically, and share a sentence describing how you would feel in this medical situation, and your thoughts about your treatment options described so far.

________________________________________________________________

| Page Break |  |
| --- | --- |

Q155
 The cardiologist prescribes therapy to control your blood pressure and lower your cholesterol. They also discuss the importance of losing weight, managing your diet, avoiding smoking, and beginning a regular but graded exercise program. If you follow this standard course of treatment, your cardiologist indicates that your risk of avoiding a heart attack is 70%.   However, it is hard to say whether your chest pain will stop even with this course of treatment, so the cardiologist recommends a new treatment known as a drug eluting stent, which would be implanted in your chest to open up the area where there is blockage. The stent would be implanted through a tube inserted through a small hole; major surgery is not required.   The Food and Drug Administration has approved the use of this new type of stent to prevent heart attacks, because stents have been shown to increase how long you live. Insertion of the stent is also likely to improve your symptoms. There are some complications following stent insertion, including the possibility that the stent itself could become blocked. The cardiologist recommends the treatment despite these possible complications.   In this case, the stent will cost an additional $55,000. Please imagine you have full health insurance, and you have already met your annual deductible. Your insurance company has a unique program, in which they cover special drugs with a cash payment, called an indemnity (similar to car insurance). Because your physician has indicated that you are a candidate for the stent, **you receive a $55,000 cash payment from your insurance company. You receive the money regardless of whether you decide to take the the stent.** You decide whether you spend the cash on the the stent or instead keep the money for whatever other purposes you may choose. In deciding, please be realistic.

Q268 On the next page you will share your decision.  First, now please write two sentences putting in your own words, (1) your understanding of the treatment options, and (2) your insurance situation, as described above.

________________________________________________________________

End of Block: CAD - VignetteC/High/Indemnity

# CAD - VignetteC/Low/Indemnity

Q99

 Over the last month, you have noticed an occasional feeling of discomfort in your chest, which generally lasts for 5-10 minutes. At first, you thought it was just indigestion, but now you notice that the feeling occurs when you climb stairs and has no relationship to food.   Concerned, you visit your primary care doctor, who does an examination, some blood tests, and an electrocardiogram (EKG). The examination shows a mildly elevated blood pressure, and the blood tests show a moderately elevated cholesterol level. The EKG is normal.   Still, concerned that your symptoms suggested the possibility of heart disease, your doctor refers you to a cardiologist (heart specialist), who recommends taking a stress echocardiogram, which means exercising on a treadmill while your heart is monitored. While exercising, the discomfort in your chest returns, and the EKG shows abnormalities in your heart function. These results strongly suggest that you have a blockage in your coronary arteries. This is confirmed with another test, a coronary angiogram, which shows one area with a lot of blockage.

Q156 Please refer to the story specifically, and share a sentence describing how you would feel in this medical situation, and your thoughts about your treatment options described so far.

________________________________________________________________

| Page Break |  |
| --- | --- |

Q157 The cardiologist prescribes therapy to control your blood pressure and lower your cholesterol. They also discuss the importance of losing weight, managing your diet, avoiding smoking, and beginning a regular but graded exercise program. If you follow this standard course of treatment, your cardiologist indicates that your risk of avoiding a heart attack is 70%.   However, it is hard to say whether your chest pain will stop even with this course of treatment, so the cardiologist recommends a new treatment known as a drug eluting stent, which would be implanted in your chest to open up the area where there is blockage. The stent would be implanted through a tube inserted through a small hole; major surgery is not required.   The Food and Drug Administration has approved the use of stents for patients who have already had heart attacks, because studies have shown that stents can help them live longer after a heart attack. Your cardiologist is also allowed to prescribe a stent “off-label” for patients who have not had a heart attack yet. But using drug eluting stents to help prevent a heart attack has not proven to help you live longer. Insertion of the stent will probably improve your symptoms, and using the stent in addition to drugs and lifestyle changes may decrease your risk for heart attack even more, but is not expected to change your long-term risk of death from heart disease. There are some complications following stent insertion, including the possibility that the stent itself could become blocked. The cardiologist recommends the treatment despite these possible complications.   In this case, the stent will cost an additional $55,000. Please imagine you have full health insurance, and you have already met your annual deductible. Your insurance company has a unique program, in which they cover special drugs with a cash payment, called an indemnity (similar to car insurance). Because your physician has indicated that you are a candidate for the stent, **you receive a $55,000 cash payment from your insurance company. You receive the money regardless of whether you decide to take the the stent.** You decide whether you spend the cash on the the stent or instead keep the money for whatever other purposes you may choose. In deciding, please be realistic.

Q267 On the next page you will share your decision.  First, now please write two sentences putting in your own words, (1) your understanding of the treatment options, and (2) your insurance situation, as described above.

________________________________________________________________

End of Block: CAD - VignetteC/Low/Indemnity

# CAD - VignetteC/High/Traditional

Q101

 Over the last month, you have noticed an occasional feeling of discomfort in your chest, which generally lasts for 5-10 minutes. At first, you thought it was just indigestion, but now you notice that the feeling occurs when you climb stairs and has no relationship to food.   Concerned, you visit your primary care doctor, who does an examination, some blood tests, and an electrocardiogram (EKG). The examination shows a mildly elevated blood pressure, and the blood tests show a moderately elevated cholesterol level. The EKG is normal.   Still, concerned that your symptoms suggested the possibility of heart disease, your doctor refers you to a cardiologist (heart specialist), who recommends taking a stress echocardiogram, which means exercising on a treadmill while your heart is monitored. While exercising, the discomfort in your chest returns, and the EKG shows abnormalities in your heart function. These results strongly suggest that you have a blockage in your coronary arteries. This is confirmed with another test, a coronary angiogram, which shows one area with a lot of blockage.

Q158 Please refer to the story specifically, and share a sentence describing how you would feel in this medical situation, and your thoughts about your treatment options described so far.

________________________________________________________________

| Page Break |  |
| --- | --- |

Q159
The cardiologist prescribes therapy to control your blood pressure and lower your cholesterol. They also discuss the importance of losing weight, managing your diet, avoiding smoking, and beginning a regular but graded exercise program. If you follow this standard course of treatment, your cardiologist indicates that your risk of avoiding a heart attack is 70%. However, it is hard to say whether your chest pain will stop even with this course of treatment, so the cardiologist recommends a new treatment known as a drug eluting stent, which would be implanted in your chest to open up the area where there is blockage. The stent would be implanted through a tube inserted through a small hole; major surgery is not required. The Food and Drug Administration has approved the use of this new type of stent to prevent heart attacks, because stents have been shown to increase how long you live. Insertion of the stent is also likely to improve your symptoms. There are some complications following stent insertion, including the possibility that the stent itself could become blocked. The cardiologist recommends the treatment despite these possible complications.  In this case, the stent will cost an additional $55,000. Please imagine **you have full health insurance**, and you have already met your annual deductible, so there is no additional cost to you out of pocket, if you choose to take the the stent treatment. In deciding, please be realistic.

Q266 On the next page you will share your decision.  First, now please write two sentences putting in your own words, (1) your understanding of the treatment options, and (2) your insurance situation, as described above.

________________________________________________________________

End of Block: CAD - VignetteC/High/Traditional

# CAD - VignetteC/Low/Traditional

Q100


 Over the last month, you have noticed an occasional feeling of discomfort in your chest, which generally lasts for 5-10 minutes. At first, you thought it was just indigestion, but now you notice that the feeling occurs when you climb stairs and has no relationship to food.   Concerned, you visit your primary care doctor, who does an examination, some blood tests, and an electrocardiogram (EKG). The examination shows a mildly elevated blood pressure, and the blood tests show a moderately elevated cholesterol level. The EKG is normal.   Still, concerned that your symptoms suggested the possibility of heart disease, your doctor refers you to a cardiologist (heart specialist), who recommends taking a stress echocardiogram, which means exercising on a treadmill while your heart is monitored. While exercising, the discomfort in your chest returns, and the EKG shows abnormalities in your heart function. These results strongly suggest that you have a blockage in your coronary arteries. This is confirmed with another test, a coronary angiogram, which shows one area with a lot of blockage.

Q160 Please refer to the story specifically, and share a sentence describing how you would feel in this medical situation, and your thoughts about your treatment options described so far.

________________________________________________________________

| Page Break |  |
| --- | --- |

Q161 The cardiologist prescribes therapy to control your blood pressure and lower your cholesterol. They also discuss the importance of losing weight, managing your diet, avoiding smoking, and beginning a regular but graded exercise program. If you follow this standard course of treatment, your cardiologist indicates that your risk of avoiding a heart attack is 70%.   However, it is hard to say whether your chest pain will stop even with this course of treatment, so the cardiologist recommends a new treatment known as a drug eluting stent, which would be implanted in your chest to open up the area where there is blockage. The stent would be implanted through a tube inserted through a small hole; major surgery is not required.   The Food and Drug Administration has approved the use of stents for patients who have already had heart attacks, because studies have shown that stents can help them live longer after a heart attack. Your cardiologist is also allowed to prescribe a stent “off-label” for patients who have not had a heart attack yet. But using drug eluting stents to help prevent a heart attack has not proven to help you live longer. Insertion of the stent will probably improve your symptoms, and using the stent in addition to drugs and lifestyle changes may decrease your risk for heart attack even more, but is not expected to change your long-term risk of death from heart disease.   In this case, the stent will cost an additional $55,000. Please imagine **you have full health insurance**, and you have already met your annual deductible, so there is no additional cost to you out of pocket, if you choose to take the the stent treatment. In deciding, please be realistic.

Q265 On the next page you will share your decision.  First, now please write two sentences putting in your own words, (1) your understanding of the treatment options, and (2) your insurance situation, as described above.

________________________________________________________________

End of Block: CAD - VignetteC/Low/Traditional

# CAD - VignetteC/Low/Uninsured

Q102


 Over the last month, you have noticed an occasional feeling of discomfort in your chest, which generally lasts for 5-10 minutes. At first, you thought it was just indigestion, but now you notice that the feeling occurs when you climb stairs and has no relationship to food.   Concerned, you visit your primary care doctor, who does an examination, some blood tests, and an electrocardiogram (EKG). The examination shows a mildly elevated blood pressure, and the blood tests show a moderately elevated cholesterol level. The EKG is normal.   Still, concerned that your symptoms suggested the possibility of heart disease, your doctor refers you to a cardiologist (heart specialist), who recommends taking a stress echocardiogram, which means exercising on a treadmill while your heart is monitored. While exercising, the discomfort in your chest returns, and the EKG shows abnormalities in your heart function. These results strongly suggest that you have a blockage in your coronary arteries. This is confirmed with another test, a coronary angiogram, which shows one area with a lot of blockage.

Q162 Please refer to the story specifically, and share a sentence describing how you would feel in this medical situation, and your thoughts about your treatment options described so far.

________________________________________________________________

| Page Break |  |
| --- | --- |

Q163 The cardiologist prescribes therapy to control your blood pressure and lower your cholesterol. They also discuss the importance of losing weight, managing your diet, avoiding smoking, and beginning a regular but graded exercise program. If you follow this standard course of treatment, your cardiologist indicates that your risk of avoiding a heart attack is 70%.   However, it is hard to say whether your chest pain will stop even with this course of treatment, so the cardiologist recommends a new treatment known as a drug eluting stent, which would be implanted in your chest to open up the area where there is blockage. The stent would be implanted through a tube inserted through a small hole; major surgery is not required.   The Food and Drug Administration has approved the use of stents for patients who have already had heart attacks, because studies have shown that stents can help them live longer after a heart attack. Your cardiologist is also allowed to prescribe a stent “off-label” for patients who have not had a heart attack yet. But using drug eluting stents to help prevent a heart attack has not proven to help you live longer. Insertion of the stent will probably improve your symptoms, and using the stent in addition to drugs and lifestyle changes may decrease your risk for heart attack even more, but is not expected to change your long-term risk of death from heart disease.    In this case, the stent will cost an additional $55,000. Please imagine that **you currently do not have health insurance**, however you do have the same amount of wealth and credit that you actually have in the real world (the amount you provided in your answer above). So, only if you have enough money available, you can pay for the the stent yourself. Think back to your answer to the purchasing power above. If you do have that much purchasing power, please consider however, other things you may prefer to do with the money. Please be realistic.

Q264 On the next page you will share your decision.  First, now please write two sentences putting in your own words, (1) your understanding of the treatment options, and (2) your insurance situation, as described above.

________________________________________________________________

End of Block: CAD - VignetteC/Low/Uninsured

# CAD - VignetteC/High/Uninsured

Q103


 Over the last month, you have noticed an occasional feeling of discomfort in your chest, which generally lasts for 5-10 minutes. At first, you thought it was just indigestion, but now you notice that the feeling occurs when you climb stairs and has no relationship to food.   Concerned, you visit your primary care doctor, who does an examination, some blood tests, and an electrocardiogram (EKG). The examination shows a mildly elevated blood pressure, and the blood tests show a moderately elevated cholesterol level. The EKG is normal.   Still, concerned that your symptoms suggested the possibility of heart disease, your doctor refers you to a cardiologist (heart specialist), who recommends taking a stress echocardiogram, which means exercising on a treadmill while your heart is monitored. While exercising, the discomfort in your chest returns, and the EKG shows abnormalities in your heart function. These results strongly suggest that you have a blockage in your coronary arteries. This is confirmed with another test, a coronary angiogram, which shows one area with a lot of blockage.

Q164 Please refer to the story specifically, and share a sentence describing how you would feel in this medical situation, and your thoughts about your treatment options described so far.

________________________________________________________________

| Page Break |  |
| --- | --- |

Q165 The cardiologist prescribes therapy to control your blood pressure and lower your cholesterol. They also discuss the importance of losing weight, managing your diet, avoiding smoking, and beginning a regular but graded exercise program. If you follow this standard course of treatment, your cardiologist indicates that your risk of avoiding a heart attack is 70%.   However, it is hard to say whether your chest pain will stop even with this course of treatment, so the cardiologist recommends a new treatment known as a drug eluting stent, which would be implanted in your chest to open up the area where there is blockage. The stent would be implanted through a tube inserted through a small hole; major surgery is not required.   The Food and Drug Administration has approved the use of this new type of stent to prevent heart attacks, because stents have been shown to increase how long you live. Insertion of the stent is also likely to improve your symptoms. There are some complications following stent insertion, including the possibility that the stent itself could become blocked. The cardiologist recommends the treatment despite these possible complications.   In this case, the stent will cost an additional $55,000. Please imagine that **you currently do not have health insurance**, however you do have the same amount of wealth and credit that you actually have in the real world (the amount you provided in your answer above). So, only if you have enough money available, you can pay for the the stent yourself. Think back to your answer to the purchasing power above. If you do have that much purchasing power, please consider however, other things you may prefer to do with the money. Please be realistic.

Q263 On the next page you will share your decision.  First, now please write two sentences putting in your own words, (1) your understanding of the treatment options, and (2) your insurance situation, as described above.

________________________________________________________________

End of Block: CAD - VignetteC/High/Uninsured

# GERD - VignetteD/High/Indemnity

Q61
 You have a long history of heartburn, particularly after eating spicy foods. Your primary care doctor refers to your situation as GERD (which stands for Gastroesophageal Reflux Disease). Over the last year, your symptoms have gotten worse, and now you also have some difficulty swallowing solid foods, a sour taste in your mouth when you have heartburn, and frequently, a sore throat with some hoarseness after the heartburn.   Antacids help control the heartburn, but they are causing diarrhea because you are taking them more often to get relief of your symptoms. Your doctor has prescribed medications to help control the symptoms, but because you have not improved as much as you would like, your doctor refers you to a specialist in stomach and intestinal problems (a gastroenterologist).   The gastroenterologist does a test to look directly at your esophagus (the tube from your mouth to your stomach) and sees severe inflammation where the esophagus connects with the stomach. This is the cause of the heartburn and other symptoms, because the inflammation allows contents from the stomach to go back up the esophagus and into your mouth. Unless the inflammation can be controlled more effectively, you will continue to have symptoms.

Q166 Please refer to the story specifically, and share a sentence describing how you would feel in this medical situation, and your thoughts about your treatment options described so far.

________________________________________________________________

| Page Break |  |
| --- | --- |

Q167 While in reasonable health, you are overweight. Your weight has increased markedly over the last year, partly because you have eaten more bland but high-calorie foods to try to control your symptoms. The gastroenterologist says that losing weight would help control the symptoms, and in particular would decrease the sour taste in your mouth after eating, along with the sore throat and hoarseness.   The gastroenterologist tells you of another option – surgery. There are many different types of surgery for this condition, but all are intended to prevent contents from the stomach going back up the esophagus. You are interested in trying this option, because you want to have immediate relief from your worsening symptoms.   You are referred to a general surgeon, who suggests a surgical procedure that is now considered the standard of care for your condition. It can be performed as an outpatient, but the surgeon prefers to do it in the hospital, since you will be put to sleep with a general anesthetic. The hospital stay will be with 2-3 days.   About three-quarters of patients who undergo surgery experience substantial relief of their symptoms. In comparison, most patients who try to lose weight without surgery often fail to lose the weight or keep it off for the longer term. Very few patients suffer side effects from surgery.  
 In this case, surgery will cost an additional $45,000. Please imagine you have full health insurance, and you have already met your annual deductible. Your insurance company has a unique program, in which they cover special drugs with a cash payment, called an indemnity (similar to car insurance). Because your physician has indicated that you are a candidate for surgery, **you receive a $45,000 cash payment from your insurance company. You receive the money regardless of whether you decide to take the surgery.** You decide whether you spend the cash on the surgery or instead keep the money for whatever other purposes you may choose. In deciding, please be realistic.

Q262 On the next page you will share your decision.  First, now please write two sentences putting in your own words, (1) your understanding of the treatment options, and (2) your insurance situation, as described above.

________________________________________________________________

End of Block: GERD - VignetteD/High/Indemnity

# GERD - VignetteD/Low/Indemnity

Q104
 You have a long history of heartburn, particularly after eating spicy foods. Your primary care doctor refers to your situation as GERD (which stands for Gastroesophageal Reflux Disease). Over the last year, your symptoms have gotten worse, and now you also have some difficulty swallowing solid foods, a sour taste in your mouth when you have heartburn, and frequently, a sore throat with some hoarseness after the heartburn.   Antacids help control the heartburn, but they are causing diarrhea because you are taking them more often to get relief of your symptoms. Your doctor has prescribed medications to help control the symptoms, but because you have not improved as much as you would like, your doctor refers you to a specialist in stomach and intestinal problems (a gastroenterologist).   The gastroenterologist does a test to look directly at your esophagus (the tube from your mouth to your stomach) and sees severe inflammation where the esophagus connects with the stomach. This is the cause of the heartburn and other symptoms, because the inflammation allows contents from the stomach to go back up the esophagus and into your mouth. Unless the inflammation can be controlled more effectively, you will continue to have symptoms.

Q168 Please refer to the story specifically, and share a sentence describing how you would feel in this medical situation, and your thoughts about your treatment options described so far.

________________________________________________________________

| Page Break |  |
| --- | --- |

Q169 While in reasonable health, you are overweight. Your weight has increased markedly over the last year, partly because you have eaten more bland but high-calorie foods to try to control your symptoms. The gastroenterologist says that losing weight would help control the symptoms, and in particular would decrease the sour taste in your mouth after eating, along with the sore throat and hoarseness.   The gastroenterologist tells you of another option – surgery. There are many different types of surgery for this condition, but all are intended to prevent contents from the stomach going back up the esophagus. You are interested in trying this option, because you want to have immediate relief from your worsening symptoms.   You are referred to a general surgeon, who suggests a surgical procedure that is now considered the standard of care for your condition. It can be performed as an outpatient, but the surgeon prefers to do it in the hospital, since you will be put to sleep with a general anesthetic. The hospital stay will be with 2-3 days.   About half of patients who have this surgery experience substantial relief of their symptoms, although results are less satisfactory in overweight people or those with poor response to the heartburn medicines they used before surgery; both of these apply to you. Side effects from surgery include bloating caused by gas build-up because of the inability to burp, and occasionally continued or more difficulty swallowing than before the surgery.   In this case, surgery will cost an additional $45,000. Please imagine you have full health insurance, and you have already met your annual deductible. Your insurance company has a unique program, in which they cover special drugs with a cash payment, called an indemnity (similar to car insurance). Because your physician has indicated that you are a candidate for surgery, **you receive a $45,000 cash payment from your insurance company. You receive the money regardless of whether you decide to take the surgery.** You decide whether you spend the cash on the surgery or instead keep the money for whatever other purposes you may choose. In deciding, please be realistic.

Q261 On the next page you will share your decision.  First, now please write two sentences putting in your own words, (1) your understanding of the treatment options, and (2) your insurance situation, as described above.

________________________________________________________________

End of Block: GERD - VignetteD/Low/Indemnity

# GERD - VignetteD/High/Traditional

Q105
 You have a long history of heartburn, particularly after eating spicy foods. Your primary care doctor refers to your situation as GERD (which stands for Gastroesophageal Reflux Disease). Over the last year, your symptoms have gotten worse, and now you also have some difficulty swallowing solid foods, a sour taste in your mouth when you have heartburn, and frequently, a sore throat with some hoarseness after the heartburn.   Antacids help control the heartburn, but they are causing diarrhea because you are taking them more often to get relief of your symptoms. Your doctor has prescribed medications to help control the symptoms, but because you have not improved as much as you would like, your doctor refers you to a specialist in stomach and intestinal problems (a gastroenterologist).   The gastroenterologist does a test to look directly at your esophagus (the tube from your mouth to your stomach) and sees severe inflammation where the esophagus connects with the stomach. This is the cause of the heartburn and other symptoms, because the inflammation allows contents from the stomach to go back up the esophagus and into your mouth. Unless the inflammation can be controlled more effectively, you will continue to have symptoms.

Q170 Please refer to the story specifically, and share a sentence describing how you would feel in this medical situation, and your thoughts about your treatment options described so far.

________________________________________________________________

| Page Break |  |
| --- | --- |

Q171 While in reasonable health, you are overweight. Your weight has increased markedly over the last year, partly because you have eaten more bland but high-calorie foods to try to control your symptoms. The gastroenterologist says that losing weight would help control the symptoms, and in particular would decrease the sour taste in your mouth after eating, along with the sore throat and hoarseness. The gastroenterologist tells you of another option – surgery. There are many different types of surgery for this condition, but all are intended to prevent contents from the stomach going back up the esophagus. You are interested in trying this option, because you want to have immediate relief from your worsening symptoms. You are referred to a general surgeon, who suggests a surgical procedure that is now considered the standard of care for your condition. It can be performed as an outpatient, but the surgeon prefers to do it in the hospital, since you will be put to sleep with a general anesthetic. The hospital stay will be with 2-3 days. About three-quarters of patients who undergo surgery experience substantial relief of their symptoms. In comparison, most patients who try to lose weight without surgery often fail to lose the weight or keep it off for the longer term. Very few patients suffer side effects from surgery. In this case, surgery will cost an additional $45,000. Please imagine **you have full health insurance**, and you have already met your annual deductible, so there is no additional cost to you out of pocket, if you choose to take the surgery treatment. In deciding, please be realistic.

Q260 On the next page you will share your decision.  First, now please write two sentences putting in your own words, (1) your understanding of the treatment options, and (2) your insurance situation, as described above.

________________________________________________________________

End of Block: GERD - VignetteD/High/Traditional

# GERD - VignetteD/Low/Traditional

Q106
 You have a long history of heartburn, particularly after eating spicy foods. Your primary care doctor refers to your situation as GERD (which stands for Gastroesophageal Reflux Disease). Over the last year, your symptoms have gotten worse, and now you also have some difficulty swallowing solid foods, a sour taste in your mouth when you have heartburn, and frequently, a sore throat with some hoarseness after the heartburn.   Antacids help control the heartburn, but they are causing diarrhea because you are taking them more often to get relief of your symptoms. Your doctor has prescribed medications to help control the symptoms, but because you have not improved as much as you would like, your doctor refers you to a specialist in stomach and intestinal problems (a gastroenterologist).   The gastroenterologist does a test to look directly at your esophagus (the tube from your mouth to your stomach) and sees severe inflammation where the esophagus connects with the stomach. This is the cause of the heartburn and other symptoms, because the inflammation allows contents from the stomach to go back up the esophagus and into your mouth. Unless the inflammation can be controlled more effectively, you will continue to have symptoms.

Q172 Please refer to the story specifically, and share a sentence describing how you would feel in this medical situation, and your thoughts about your treatment options described so far.

________________________________________________________________

| Page Break |  |
| --- | --- |

Q173 While in reasonable health, you are overweight. Your weight has increased markedly over the last year, partly because you have eaten more bland but high-calorie foods to try to control your symptoms. The gastroenterologist says that losing weight would help control the symptoms, and in particular would decrease the sour taste in your mouth after eating, along with the sore throat and hoarseness.   The gastroenterologist tells you of another option – surgery. There are many different types of surgery for this condition, but all are intended to prevent contents from the stomach going back up the esophagus. You are interested in trying this option, because you want to have immediate relief from your worsening symptoms.   You are referred to a general surgeon, who suggests a surgical procedure that is now considered the standard of care for your condition. It can be performed as an outpatient, but the surgeon prefers to do it in the hospital, since you will be put to sleep with a general anesthetic. The hospital stay will be with 2-3 days.   About half of patients who have this surgery experience substantial relief of their symptoms, although results are less satisfactory in overweight people or those with poor response to the heartburn medicines they used before surgery; both of these apply to you. Side effects from surgery include bloating caused by gas build-up because of the inability to burp, and occasionally continued or more difficulty swallowing than before the surgery.   In this case, surgery will cost an additional $45,000. Please imagine **you have full health insurance**, and you have already met your annual deductible, so there is no additional cost to you out of pocket, if you choose to take the surgery treatment. In deciding, please be realistic.

Q259 On the next page you will share your decision.  First, now please write two sentences putting in your own words, (1) your understanding of the treatment options, and (2) your insurance situation, as described above.

________________________________________________________________

End of Block: GERD - VignetteD/Low/Traditional

# GERD - VignetteD/Low/Uninsured

Q108
 You have a long history of heartburn, particularly after eating spicy foods. Your primary care doctor refers to your situation as GERD (which stands for Gastroesophageal Reflux Disease). Over the last year, your symptoms have gotten worse, and now you also have some difficulty swallowing solid foods, a sour taste in your mouth when you have heartburn, and frequently, a sore throat with some hoarseness after the heartburn.   Antacids help control the heartburn, but they are causing diarrhea because you are taking them more often to get relief of your symptoms. Your doctor has prescribed medications to help control the symptoms, but because you have not improved as much as you would like, your doctor refers you to a specialist in stomach and intestinal problems (a gastroenterologist).   The gastroenterologist does a test to look directly at your esophagus (the tube from your mouth to your stomach) and sees severe inflammation where the esophagus connects with the stomach. This is the cause of the heartburn and other symptoms, because the inflammation allows contents from the stomach to go back up the esophagus and into your mouth. Unless the inflammation can be controlled more effectively, you will continue to have symptoms.

Q174 Please refer to the story specifically, and share a sentence describing how you would feel in this medical situation, and your thoughts about your treatment options described so far.

________________________________________________________________

| Page Break |  |
| --- | --- |

Q175 While in reasonable health, you are overweight. Your weight has increased markedly over the last year, partly because you have eaten more bland but high-calorie foods to try to control your symptoms. The gastroenterologist says that losing weight would help control the symptoms, and in particular would decrease the sour taste in your mouth after eating, along with the sore throat and hoarseness.   The gastroenterologist tells you of another option – surgery. There are many different types of surgery for this condition, but all are intended to prevent contents from the stomach going back up the esophagus. You are interested in trying this option, because you want to have immediate relief from your worsening symptoms.   You are referred to a general surgeon, who suggests a surgical procedure that is now considered the standard of care for your condition. It can be performed as an outpatient, but the surgeon prefers to do it in the hospital, since you will be put to sleep with a general anesthetic. The hospital stay will be with 2-3 days.   About half of patients who have this surgery experience substantial relief of their symptoms, although results are less satisfactory in overweight people or those with poor response to the heartburn medicines they used before surgery; both of these apply to you. Side effects from surgery include bloating caused by gas build-up because of the inability to burp, and occasionally continued or more difficulty swallowing than before the surgery.   In this case, surgery will cost an additional $45,000. Please imagine that **you currently do not have health insurance**, however you do have the same amount of wealth and credit that you actually have in the real world (the amount you provided in your answer above). So, only if you have enough money available, you can pay for the surgery yourself. Think back to your answer to the purchasing power above. If you do have that much purchasing power, please consider however, other things you may prefer to do with the money. Please be realistic.

Q258 On the next page you will share your decision.  First, now please write two sentences putting in your own words, (1) your understanding of the treatment options, and (2) your insurance situation, as described above.

________________________________________________________________

End of Block: GERD - VignetteD/Low/Uninsured

# GERD - VignetteD/High/Uninsured

Q107
 You have a long history of heartburn, particularly after eating spicy foods. Your primary care doctor refers to your situation as GERD (which stands for Gastroesophageal Reflux Disease). Over the last year, your symptoms have gotten worse, and now you also have some difficulty swallowing solid foods, a sour taste in your mouth when you have heartburn, and frequently, a sore throat with some hoarseness after the heartburn.   Antacids help control the heartburn, but they are causing diarrhea because you are taking them more often to get relief of your symptoms. Your doctor has prescribed medications to help control the symptoms, but because you have not improved as much as you would like, your doctor refers you to a specialist in stomach and intestinal problems (a gastroenterologist).   The gastroenterologist does a test to look directly at your esophagus (the tube from your mouth to your stomach) and sees severe inflammation where the esophagus connects with the stomach. This is the cause of the heartburn and other symptoms, because the inflammation allows contents from the stomach to go back up the esophagus and into your mouth. Unless the inflammation can be controlled more effectively, you will continue to have symptoms.

Q176 Please refer to the story specifically, and share a sentence describing how you would feel in this medical situation, and your thoughts about your treatment options described so far.

________________________________________________________________

| Page Break |  |
| --- | --- |

Q177 While in reasonable health, you are overweight. Your weight has increased markedly over the last year, partly because you have eaten more bland but high-calorie foods to try to control your symptoms. The gastroenterologist says that losing weight would help control the symptoms, and in particular would decrease the sour taste in your mouth after eating, along with the sore throat and hoarseness. The gastroenterologist tells you of another option – surgery. There are many different types of surgery for this condition, but all are intended to prevent contents from the stomach going back up the esophagus. You are interested in trying this option, because you want to have immediate relief from your worsening symptoms. You are referred to a general surgeon, who suggests a surgical procedure that is now considered the standard of care for your condition. It can be performed as an outpatient, but the surgeon prefers to do it in the hospital, since you will be put to sleep with a general anesthetic. The hospital stay will be with 2-3 days. About three-quarters of patients who undergo surgery experience substantial relief of their symptoms. In comparison, most patients who try to lose weight without surgery often fail to lose the weight or keep it off for the longer term. Very few patients suffer side effects from surgery. In this case, surgery will cost an additional $45,000. Please imagine that **you currently do not have health insurance**, however you do have the same amount of wealth and credit that you actually have in the real world (the amount you provided in your answer above). So, only if you have enough money available, you can pay for the surgery yourself. Think back to your answer to the purchasing power above. If you do have that much purchasing power, please consider however, other things you may prefer to do with the money. Please be realistic.

Q257 On the next page you will share your decision.  First, now please write two sentences putting in your own words, (1) your understanding of the treatment options, and (2) your insurance situation, as described above.

________________________________________________________________

End of Block: GERD - VignetteD/High/Uninsured

# AMD - VignetteE/Low/Uninsured

Q113
 Your vision has been gradually getting worse over the last several years and is now starting to limit your ability to read, drive, and do other daily activities. You have not seen an eye doctor about this before, because you have been doing well enough, and have a general dislike of seeing doctors. You mention your worsening eyesight to your primary care doctor, who refers you to an ophthalmologist (specialist eye doctor).   The eye doctor examines you and makes the diagnosis of age-related macular degeneration (AMD). This is a common condition with aging, and it will get worse unless treated. You could even become legally blind in just a few years. The eye doctor says that there is now an excellent drug (Prexilin) to treat your type of AMD. The drug must be injected into the eyes, but this is mostly painless and easy to tolerate.   Treatment with Prexilin typically slows or stops the progression of vision loss, and in about 1/3 of cases, some improvement in vision occurs. You will have injections every month for 3 or 4 months, but if a lot of improvement is noted, the shots will be less often. The eye doctor describes other forms of treatment for AMD, including laser therapy, but believes you should start with Prexilin alone.

Q178 Please refer to the story specifically, and share a sentence describing how you would feel in this medical situation, and your thoughts about your treatment options described so far.

________________________________________________________________

| Page Break |  |
| --- | --- |

Q179 Not long after you see the eye doctor, you talk to your older brother who you find out had also been diagnosed with this eye disease several years earlier. He is also getting direct injections into his eyes, but with a different drug (Retinax). He is very happy with the results, because his vision got better very quickly after starting the treatments, and it is still better than it was before he started the treatment.   You are curious about Retinax, and on a repeat visit to your eye doctor, you ask him about the difference between the two drugs. He tells you that both Prelixin and Retinax have been shown in many clinical studies to be effective for stopping the AMD from getting worse. Retinax is much newer than Prexilin. Also, Retinax costs a lot more, and there is less real-world data about its risks and benefits, so the eye doctor recommends sticking with Prexilin.  
 In this case, Retinax will cost an additional $15,000. Please imagine that **you currently do not have health insurance**, however you do have the same amount of wealth and credit that you actually have in the real world (the amount you provided in your answer above). So, only if you have enough money available, you can pay for the Retinax yourself. Think back to your answer to the purchasing power above. If you do have that much purchasing power, please consider however, other things you may prefer to do with the money. Please be realistic.

Q256 On the next page you will share your decision.  First, now please write two sentences putting in your own words, (1) your understanding of the treatment options, and (2) your insurance situation, as described above.

________________________________________________________________

End of Block: AMD - VignetteE/Low/Uninsured

# AMD - VignetteE/High/Uninsured

Q112
 Your vision has been gradually getting worse over the last several years and is now starting to limit your ability to read, drive, and do other daily activities. You have not seen an eye doctor about this before, because you have been doing well enough, and have a general dislike of seeing doctors. You mention your worsening eyesight to your primary care doctor, who refers you to an ophthalmologist (specialist eye doctor).   The eye doctor examines you and makes the diagnosis of age-related macular degeneration (AMD). This is a common condition with aging, and it will get worse unless treated. You could even become legally blind in just a few years. The eye doctor says that there is now an excellent drug (Prexilin) to treat your type of AMD. The drug must be injected into the eyes, but this is mostly painless and easy to tolerate.   Treatment with Prexilin typically slows or stops the progression of vision loss, and in about 1/3 of cases, some improvement in vision occurs. You will have injections every month for 3 or 4 months, but if a lot of improvement is noted, the shots will be less often. The eye doctor describes other forms of treatment for AMD, including laser therapy, but believes you should start with Prexilin alone.  

Q180 Please refer to the story specifically, and share a sentence describing how you would feel in this medical situation, and your thoughts about your treatment options described so far.

________________________________________________________________

| Page Break |  |
| --- | --- |

Q181 Not long after you see the eye doctor, you talk to your older brother who you find out had also been diagnosed with this eye disease several years earlier. He is also getting direct injections into his eyes, but with a different drug (Retinax). He is very happy with the results, because his vision got better very quickly after starting the treatments, and it is still better than it was before he started the treatment.   You are curious about Retinax, and on a repeat visit to your eye doctor, you ask him about the difference between the two drugs. He tells you that both Prelixin and Retinax have been shown in many clinical studies to be effective for stopping the AMD from getting worse. Retinax costs a lot more than Prexilin. Although the drugs have not been tested in head-to-head trials, the data suggests that Retinax improves vision in 2/3 of cases, rather than only 1/3 of cases like Prexilin. Even so, the eye doctor explains that he is more familiar with Prexilin, because he is a consultant for the company.   In this case, Retinax will cost an additional $15,000. Please imagine that **you currently do not have health insurance,** however you do have the same amount of wealth and credit that you actually have in the real world (the amount you provided in your answer above). So, only if you have enough money available, you can pay for the Retinax yourself. Think back to your answer to the purchasing power above. If you do have that much purchasing power, please consider however, other things you may prefer to do with the money. Please be realistic.

Q255 On the next page you will share your decision.  First, now please write two sentences putting in your own words, (1) your understanding of the treatment options, and (2) your insurance situation, as described above.

________________________________________________________________

End of Block: AMD - VignetteE/High/Uninsured

# AMD - VignetteE/Low/Traditional

Q111
 Your vision has been gradually getting worse over the last several years and is now starting to limit your ability to read, drive, and do other daily activities. You have not seen an eye doctor about this before, because you have been doing well enough, and have a general dislike of seeing doctors. You mention your worsening eyesight to your primary care doctor, who refers you to an ophthalmologist (specialist eye doctor).   The eye doctor examines you and makes the diagnosis of age-related macular degeneration (AMD). This is a common condition with aging, and it will get worse unless treated. You could even become legally blind in just a few years. The eye doctor says that there is now an excellent drug (Prexilin) to treat your type of AMD. The drug must be injected into the eyes, but this is mostly painless and easy to tolerate.   Treatment with Prexilin typically slows or stops the progression of vision loss, and in about 1/3 of cases, some improvement in vision occurs. You will have injections every month for 3 or 4 months, but if a lot of improvement is noted, the shots will be less often. The eye doctor describes other forms of treatment for AMD, including laser therapy, but believes you should start with Prexilin alone.  

Q182 Please refer to the story specifically, and share a sentence describing how you would feel in this medical situation, and your thoughts about your treatment options described so far.

________________________________________________________________

| Page Break |  |
| --- | --- |

Q183 Not long after you see the eye doctor, you talk to your older brother who you find out had also been diagnosed with this eye disease several years earlier. He is also getting direct injections into his eyes, but with a different drug (Retinax). He is very happy with the results, because his vision got better very quickly after starting the treatments, and it is still better than it was before he started the treatment.   You are curious about Retinax, and on a repeat visit to your eye doctor, you ask him about the difference between the two drugs. He tells you that both Prelixin and Retinax have been shown in many clinical studies to be effective for stopping the AMD from getting worse. Retinax is much newer than Prexilin. Also, Retinax costs a lot more, and there is less real-world data about its risks and benefits, so the eye doctor recommends sticking with Prexilin.   In this case, Retinax will cost an additional $15,000. Please imagine **you have full health insurance**, and you have already met your annual deductible, so there is no additional cost to you out of pocket, if you choose to take the Retinax treatment. In deciding, please be realistic.

Q254 On the next page you will share your decision.  First, now please write two sentences putting in your own words, (1) your understanding of the treatment options, and (2) your insurance situation, as described above.

________________________________________________________________

End of Block: AMD - VignetteE/Low/Traditional

# AMD - VignetteE/High/Traditional

Q110
 Your vision has been gradually getting worse over the last several years and is now starting to limit your ability to read, drive, and do other daily activities. You have not seen an eye doctor about this before, because you have been doing well enough, and have a general dislike of seeing doctors. You mention your worsening eyesight to your primary care doctor, who refers you to an ophthalmologist (specialist eye doctor).   The eye doctor examines you and makes the diagnosis of age-related macular degeneration (AMD). This is a common condition with aging, and it will get worse unless treated. You could even become legally blind in just a few years. The eye doctor says that there is now an excellent drug (Prexilin) to treat your type of AMD. The drug must be injected into the eyes, but this is mostly painless and easy to tolerate.   Treatment with Prexilin typically slows or stops the progression of vision loss, and in about 1/3 of cases, some improvement in vision occurs. You will have injections every month for 3 or 4 months, but if a lot of improvement is noted, the shots will be less often. The eye doctor describes other forms of treatment for AMD, including laser therapy, but believes you should start with Prexilin alone.

Q184 Please refer to the story specifically, and share a sentence describing how you would feel in this medical situation, and your thoughts about your treatment options described so far.

________________________________________________________________

| Page Break |  |
| --- | --- |

Q185 Not long after you see the eye doctor, you talk to your older brother who you find out had also been diagnosed with this eye disease several years earlier. He is also getting direct injections into his eyes, but with a different drug (Retinax). He is very happy with the results, because his vision got better very quickly after starting the treatments, and it is still better than it was before he started the treatment.   You are curious about Retinax, and on a repeat visit to your eye doctor, you ask him about the difference between the two drugs. He tells you that both Prelixin and Retinax have been shown in many clinical studies to be effective for stopping the AMD from getting worse. Retinax costs a lot more than Prexilin. Although the drugs have not been tested in head-to-head trials, the data suggests that Retinax improves vision in 2/3 of cases, rather than only 1/3 of cases like Prexilin. Even so, the eye doctor explains that he is more familiar with Prexilin, because he is a consultant for the company.   In this case, Retinax will cost an additional $15,000. Please imagine **you have full health insurance**, and you have already met your annual deductible, so there is no additional cost to you out of pocket, if you choose to take the Retinax treatment. In deciding, please be realistic.

Q253 On the next page you will share your decision.  First, now please write two sentences putting in your own words, (1) your understanding of the treatment options, and (2) your insurance situation, as described above.

________________________________________________________________

End of Block: AMD - VignetteE/High/Traditional

# AMD - VignetteE/Low/Indemnity

Q109
 Your vision has been gradually getting worse over the last several years and is now starting to limit your ability to read, drive, and do other daily activities. You have not seen an eye doctor about this before, because you have been doing well enough, and have a general dislike of seeing doctors. You mention your worsening eyesight to your primary care doctor, who refers you to an ophthalmologist (specialist eye doctor).   The eye doctor examines you and makes the diagnosis of age-related macular degeneration (AMD). This is a common condition with aging, and it will get worse unless treated. You could even become legally blind in just a few years. The eye doctor says that there is now an excellent drug (Prexilin) to treat your type of AMD. The drug must be injected into the eyes, but this is mostly painless and easy to tolerate.   Treatment with Prexilin typically slows or stops the progression of vision loss, and in about 1/3 of cases, some improvement in vision occurs. You will have injections every month for 3 or 4 months, but if a lot of improvement is noted, the shots will be less often. The eye doctor describes other forms of treatment for AMD, including laser therapy, but believes you should start with Prexilin alone.

Q186 Please refer to the story specifically, and share a sentence describing how you would feel in this medical situation, and your thoughts about your treatment options described so far.

________________________________________________________________

| Page Break |  |
| --- | --- |

Q187 Not long after you see the eye doctor, you talk to your older brother who you find out had also been diagnosed with this eye disease several years earlier. He is also getting direct injections into his eyes, but with a different drug (Retinax). He is very happy with the results, because his vision got better very quickly after starting the treatments, and it is still better than it was before he started the treatment.   You are curious about Retinax, and on a repeat visit to your eye doctor, you ask him about the difference between the two drugs. He tells you that both Prelixin and Retinax have been shown in many clinical studies to be effective for stopping the AMD from getting worse. Retinax is much newer than Prexilin. Also, Retinax costs a lot more, and there is less real-world data about its risks and benefits, so the eye doctor recommends sticking with Prexilin.    In this case, Retinax will cost an additional $15,000. Please imagine you have full health insurance, and you have already met your annual deductible. Your insurance company has a unique program, in which they cover special drugs with a cash payment, called an indemnity (similar to car insurance). Because your physician has indicated that you are a candidate for Retinax, **you receive a $15,000 cash payment from your insurance company. You receive the money regardless of whether you decide to take the Retinax**. You decide whether you spend the cash on the Retinax or instead keep the money for whatever other purposes you may choose. In deciding, please be realistic.

Q252 On the next page you will share your decision.  First, now please write two sentences putting in your own words, (1) your understanding of the treatment options, and (2) your insurance situation, as described above.

________________________________________________________________

End of Block: AMD - VignetteE/Low/Indemnity

# AMD - VignetteE/High/Indemnity

Q62 Your vision has been gradually getting worse over the last several years and is now starting to limit your ability to read, drive, and do other daily activities. You have not seen an eye doctor about this before, because you have been doing well enough, and have a general dislike of seeing doctors. You mention your worsening eyesight to your primary care doctor, who refers you to an ophthalmologist (specialist eye doctor).   The eye doctor examines you and makes the diagnosis of age-related macular degeneration (AMD). This is a common condition with aging, and it will get worse unless treated. You could even become legally blind in just a few years. The eye doctor says that there is now an excellent drug (Prexilin) to treat your type of AMD. The drug must be injected into the eyes, but this is mostly painless and easy to tolerate.   Treatment with Prexilin typically slows or stops the progression of vision loss, and in about 1/3 of cases, some improvement in vision occurs. You will have injections every month for 3 or 4 months, but if a lot of improvement is noted, the shots will be less often. The eye doctor describes other forms of treatment for AMD, including laser therapy, but believes you should start with Prexilin alone.  

Q188 Please refer to the story specifically, and share a sentence describing how you would feel in this medical situation, and your thoughts about your treatment options described so far.

________________________________________________________________

| Page Break |  |
| --- | --- |

Q189 Not long after you see the eye doctor, you talk to your older brother who you find out had also been diagnosed with this eye disease several years earlier. He is also getting direct injections into his eyes, but with a different drug (Retinax). He is very happy with the results, because his vision got better very quickly after starting the treatments, and it is still better than it was before he started the treatment.   You are curious about Retinax, and on a repeat visit to your eye doctor, you ask him about the difference between the two drugs. He tells you that both Prelixin and Retinax have been shown in many clinical studies to be effective for stopping the AMD from getting worse. Retinax costs a lot more than Prexilin. Although the drugs have not been tested in head-to-head trials, the data suggests that Retinax improves vision in 2/3 of cases, rather than only 1/3 of cases like Prexilin. Even so, the eye doctor explains that he is more familiar with Prexilin, because he is a consultant for the company.   In this case, Retinax will cost an additional $15,000. Please imagine you have full health insurance, and you have already met your annual deductible. Your insurance company has a unique program, in which they cover special drugs with a cash payment, called an indemnity (similar to car insurance). Because your physician has indicated that you are a candidate for Retinax, **you receive a $15,000 cash payment from your insurance company. You receive the money regardless of whether you decide to take the Retinax.** You decide whether you spend the cash on the Retinax or instead keep the money for whatever other purposes you may choose. In deciding, please be realistic.

Q251 On the next page you will share your decision.  First, now please write two sentences putting in your own words, (1) your understanding of the treatment options, and (2) your insurance situation, as described above.

________________________________________________________________

End of Block: AMD - VignetteE/High/Indemnity

# Psoriasis - VignetteF/High/Indemnity

Q63 You have a history of mild psoriasis (so-RI-a-sis) limited to your skin. This causes raised, scaly, red areas on your knees, elbows and scalp. You have not had any arthritis, which sometimes comes with this skin disease. You have tried many different treatments for this disease, most of which have made it better for a little while, but nothing has really lasted.   You have been pretty stressed out for several months, which has caused a flare up of your psoriasis. In the past, these flare ups have been managed with short courses of more aggressive standard therapy for your disease. Because your family, friends and co-workers are familiar with these occasional changes in your skin problems, you have not been concerned when they occur.   Your high school reunion is scheduled in four months. You did not have psoriasis in high school and will be embarrassed to attend the reunion if psoriasis is noticeable. You think about not even going. You need to make travel arrangements soon if you plan to attend, but know from experience that you cannot predict how active your psoriasis will be four months from now. In addition, the anxiety about attending will probably make the skin disease worse.  

Q190 Please refer to the story specifically, and share a sentence describing how you would feel in this medical situation, and your thoughts about your treatment options described so far.

________________________________________________________________

| Page Break |  |
| --- | --- |

Q191 You approach your dermatologist (skin doctor) about one of the newer “biologic drugs,” (Varatril), that you have seen advertised on television. The testimonials from the people in the ads is convincing, because some of them are well-known celebrities, and not just actors in commercials. You have done some reading online and believe Varatril would give you the best chance for good results.   Your dermatologist warns you that Varatril is expensive. About two thirds of patients who take the drug report that their skin has cleared. The dermatologist also explains that the side effects can be serious, since the drug interferes with the normal function of your body’s defenses against infection, cancer and other serious diseases. The dermatologist acknowledges that your dosage of Varatril would be relatively low, so such severe side effects would be less likely to occur. You just need to monitor your health carefully and avoid unnecessary risks.   The dermatologist finally agrees to prescribe Varatril if you are willing to take and completely understand the risks. The dermatologist suggests that you stop taking Varatril after the reunion, but also knows from previous experience that you will want to continue taking the drug if the psoriasis goes away completely.   In this case, Varatril will cost an additional $20,000. Please imagine you have full health insurance, and you have already met your annual deductible. Your insurance company has a unique program, in which they cover special drugs with a cash payment, called an indemnity (similar to car insurance). Because your physician has indicated that you are a candidate for Varatril, **you receive a $20,000 cash payment from your insurance company. You receive the money regardless of whether you decide to take the Varatril.** You decide whether you spend the cash on the Varatril or instead keep the money for whatever other purposes you may choose. In deciding, please be realistic.

Q250 On the next page you will share your decision.  First, now please write two sentences putting in your own words, (1) your understanding of the treatment options, and (2) your insurance situation, as described above.

________________________________________________________________

End of Block: Psoriasis - VignetteF/High/Indemnity

# Psoriasis - VignetteF/Low/Uninsured

Q115 You have a history of mild psoriasis (so-RI-a-sis) limited to your skin. This causes raised, scaly, red areas on your knees, elbows and scalp. You have not had any arthritis, which sometimes comes with this skin disease. You have tried many different treatments for this disease, most of which have made it better for a little while, but nothing has really lasted.   You have been pretty stressed out for several months, which has caused a flare up of your psoriasis. In the past, these flare ups have been managed with short courses of more aggressive standard therapy for your disease. Because your family, friends and co-workers are familiar with these occasional changes in your skin problems, you have not been concerned when they occur.   Your high school reunion is scheduled in four months. You did not have psoriasis in high school and will be embarrassed to attend the reunion if psoriasis is noticeable. You think about not even going. You need to make travel arrangements soon if you plan to attend, but know from experience that you cannot predict how active your psoriasis will be four months from now. In addition, the anxiety about attending will probably make the skin disease worse.  

Q192 Please refer to the story specifically, and share a sentence describing how you would feel in this medical situation, and your thoughts about your treatment options described so far.

________________________________________________________________

| Page Break |  |
| --- | --- |

Q193 ​You approach your dermatologist (skin doctor) about one of the newer “biologic drugs,” (Varatril), that you have seen advertised on television. The testimonials from the people in the ads is convincing, because some of them are well-known celebrities, and not just actors in commercials. You have done some reading online and believe Varatril would give you the best chance for good results.   Your dermatologist warns you that Varatril is expensive. About one third of patients who take the drug report that their skin has cleared. The dermatologist also explains that the side effects can be severe and potentially life threatening, since the drug interferes with the normal function of your body’s defenses against infection, cancer and other serious diseases.   The dermatologist finally agrees to prescribe Varatril if you are willing to take and completely understand the risks. The dermatologist suggests that you stop taking Varatril after the reunion, but also knows from previous experience that you will want to continue taking the drug if the psoriasis goes away completely.   In this case, Varatril will cost an additional $20,000. Please imagine that **you currently do not have health insurance**, however you do have the same amount of wealth and credit that you actually have in the real world (the amount you provided in your answer above). So, only if you have enough money available, you can pay for the Varatril yourself. Think back to your answer to the purchasing power above. If you do have that much purchasing power, please consider however, other things you may prefer to do with the money. Please be realistic.

Q249 On the next page you will share your decision.  First, now please write two sentences putting in your own words, (1) your understanding of the treatment options, and (2) your insurance situation, as described above.

________________________________________________________________

End of Block: Psoriasis - VignetteF/Low/Uninsured

# Psoriasis - VignetteF/High/Uninsured

Q118 You have a history of mild psoriasis (so-RI-a-sis) limited to your skin. This causes raised, scaly, red areas on your knees, elbows and scalp. You have not had any arthritis, which sometimes comes with this skin disease. You have tried many different treatments for this disease, most of which have made it better for a little while, but nothing has really lasted.   You have been pretty stressed out for several months, which has caused a flare up of your psoriasis. In the past, these flare ups have been managed with short courses of more aggressive standard therapy for your disease. Because your family, friends and co-workers are familiar with these occasional changes in your skin problems, you have not been concerned when they occur.   Your high school reunion is scheduled in four months. You did not have psoriasis in high school and will be embarrassed to attend the reunion if psoriasis is noticeable. You think about not even going. You need to make travel arrangements soon if you plan to attend, but know from experience that you cannot predict how active your psoriasis will be four months from now. In addition, the anxiety about attending will probably make the skin disease worse.

Q194 Please refer to the story specifically, and share a sentence describing how you would feel in this medical situation, and your thoughts about your treatment options described so far.

________________________________________________________________

| Page Break |  |
| --- | --- |

Q195 You approach your dermatologist (skin doctor) about one of the newer “biologic drugs,” (Varatril), that you have seen advertised on television. The testimonials from the people in the ads is convincing, because some of them are well-known celebrities, and not just actors in commercials. You have done some reading online and believe Varatril would give you the best chance for good results. Your dermatologist warns you that Varatril is expensive. About two thirds of patients who take the drug report that their skin has cleared. The dermatologist also explains that the side effects can be serious, since the drug interferes with the normal function of your body’s defenses against infection, cancer and other serious diseases. The dermatologist acknowledges that your dosage of Varatril would be relatively low, so such severe side effects would be less likely to occur. You just need to monitor your health carefully and avoid unnecessary risks. The dermatologist finally agrees to prescribe Varatril if you are willing to take and completely understand the risks. The dermatologist suggests that you stop taking Varatril after the reunion, but also knows from previous experience that you will want to continue taking the drug if the psoriasis goes away completely. In this case, Varatril will cost an additional $20,000. Please imagine that **you currently do not have health insurance**, however you do have the same amount of wealth and credit that you actually have in the real world (the amount you provided in your answer above). So, only if you have enough money available, you can pay for the Varatril yourself. Think back to your answer to the purchasing power above. If you do have that much purchasing power, please consider however, other things you may prefer to do with the money. Please be realistic.

Q248 On the next page you will share your decision.  First, now please write two sentences putting in your own words, (1) your understanding of the treatment options, and (2) your insurance situation, as described above.

________________________________________________________________

End of Block: Psoriasis - VignetteF/High/Uninsured

# Psoriasis - VignetteF/Low/Traditional

Q117
 You have a history of mild psoriasis (so-RI-a-sis) limited to your skin. This causes raised, scaly, red areas on your knees, elbows and scalp. You have not had any arthritis, which sometimes comes with this skin disease. You have tried many different treatments for this disease, most of which have made it better for a little while, but nothing has really lasted.   You have been pretty stressed out for several months, which has caused a flare up of your psoriasis. In the past, these flare ups have been managed with short courses of more aggressive standard therapy for your disease. Because your family, friends and co-workers are familiar with these occasional changes in your skin problems, you have not been concerned when they occur.   Your high school reunion is scheduled in four months. You did not have psoriasis in high school and will be embarrassed to attend the reunion if psoriasis is noticeable. You think about not even going. You need to make travel arrangements soon if you plan to attend, but know from experience that you cannot predict how active your psoriasis will be four months from now. In addition, the anxiety about attending will probably make the skin disease worse.

Q197 Please refer to the story specifically, and share a sentence describing how you would feel in this medical situation, and your thoughts about your treatment options described so far.

________________________________________________________________

| Page Break |  |
| --- | --- |

Q198 You approach your dermatologist (skin doctor) about one of the newer “biologic drugs,” (Varatril), that you have seen advertised on television. The testimonials from the people in the ads is convincing, because some of them are well-known celebrities, and not just actors in commercials. You have done some reading online and believe Varatril would give you the best chance for good results.   Your dermatologist warns you that Varatril is expensive. About one third of patients who take the drug report that their skin has cleared. The dermatologist also explains that the side effects can be severe and potentially life threatening, since the drug interferes with the normal function of your body’s defenses against infection, cancer and other serious diseases.   The dermatologist finally agrees to prescribe Varatril if you are willing to take and completely understand the risks. The dermatologist suggests that you stop taking Varatril after the reunion, but also knows from previous experience that you will want to continue taking the drug if the psoriasis goes away completely.   In this case, Varatril will cost an additional $20,000. Please imagine **you have full health insurance**, and you have already met your annual deductible, so there is no additional cost to you out of pocket, if you choose to take the Varatril treatment. In deciding, please be realistic.

Q247 On the next page you will share your decision.  First, now please write two sentences putting in your own words, (1) your understanding of the treatment options, and (2) your insurance situation, as described above.

________________________________________________________________

End of Block: Psoriasis - VignetteF/Low/Traditional

# Psoriasis - VignetteF/High/Traditional

Q116
 You have a history of mild psoriasis (so-RI-a-sis) limited to your skin. This causes raised, scaly, red areas on your knees, elbows and scalp. You have not had any arthritis, which sometimes comes with this skin disease. You have tried many different treatments for this disease, most of which have made it better for a little while, but nothing has really lasted.   You have been pretty stressed out for several months, which has caused a flare up of your psoriasis. In the past, these flare ups have been managed with short courses of more aggressive standard therapy for your disease. Because your family, friends and co-workers are familiar with these occasional changes in your skin problems, you have not been concerned when they occur.   Your high school reunion is scheduled in four months. You did not have psoriasis in high school and will be embarrassed to attend the reunion if psoriasis is noticeable. You think about not even going. You need to make travel arrangements soon if you plan to attend, but know from experience that you cannot predict how active your psoriasis will be four months from now. In addition, the anxiety about attending will probably make the skin disease worse.  

Q199 Please refer to the story specifically, and share a sentence describing how you would feel in this medical situation, and your thoughts about your treatment options described so far.

________________________________________________________________

| Page Break |  |
| --- | --- |

Q200 You approach your dermatologist (skin doctor) about one of the newer “biologic drugs,” (Varatril), that you have seen advertised on television. The testimonials from the people in the ads is convincing, because some of them are well-known celebrities, and not just actors in commercials. You have done some reading online and believe Varatril would give you the best chance for good results.   Your dermatologist warns you that Varatril is expensive. About two thirds of patients who take the drug report that their skin has cleared. The dermatologist also explains that the side effects can be serious, since the drug interferes with the normal function of your body’s defenses against infection, cancer and other serious diseases. The dermatologist acknowledges that your dosage of Varatril would be relatively low, so such severe side effects would be less likely to occur. You just need to monitor your health carefully and avoid unnecessary risks.   The dermatologist finally agrees to prescribe Varatril if you are willing to take and completely understand the risks. The dermatologist suggests that you stop taking Varatril after the reunion, but also knows from previous experience that you will want to continue taking the drug if the psoriasis goes away completely.   In this case, Varatril will cost an additional $20,000. Please imagine **you have full health insurance**, and you have already met your annual deductible, so there is no additional cost to you out of pocket, if you choose to take the Varatril treatment. In deciding, please be realistic.

Q246 On the next page you will share your decision.  First, now please write two sentences putting in your own words, (1) your understanding of the treatment options, and (2) your insurance situation, as described above.

________________________________________________________________

End of Block: Psoriasis - VignetteF/High/Traditional

# Psoriasis - VignetteF/Low/Indemnity

Q114
 You have a history of mild psoriasis (so-RI-a-sis) limited to your skin. This causes raised, scaly, red areas on your knees, elbows and scalp. You have not had any arthritis, which sometimes comes with this skin disease. You have tried many different treatments for this disease, most of which have made it better for a little while, but nothing has really lasted.   You have been pretty stressed out for several months, which has caused a flare up of your psoriasis. In the past, these flare ups have been managed with short courses of more aggressive standard therapy for your disease. Because your family, friends and co-workers are familiar with these occasional changes in your skin problems, you have not been concerned when they occur.   Your high school reunion is scheduled in four months. You did not have psoriasis in high school and will be embarrassed to attend the reunion if psoriasis is noticeable. You think about not even going. You need to make travel arrangements soon if you plan to attend, but know from experience that you cannot predict how active your psoriasis will be four months from now. In addition, the anxiety about attending will probably make the skin disease worse.  

Q201 Please refer to the story specifically, and share a sentence describing how you would feel in this medical situation, and your thoughts about your treatment options described so far.

________________________________________________________________

| Page Break |  |
| --- | --- |

Q202 You approach your dermatologist (skin doctor) about one of the newer “biologic drugs,” (Varatril), that you have seen advertised on television. The testimonials from the people in the ads is convincing, because some of them are well-known celebrities, and not just actors in commercials. You have done some reading online and believe Varatril would give you the best chance for good results.   Your dermatologist warns you that Varatril is expensive. About one third of patients who take the drug report that their skin has cleared. The dermatologist also explains that the side effects can be severe and potentially life threatening, since the drug interferes with the normal function of your body’s defenses against infection, cancer and other serious diseases.   The dermatologist finally agrees to prescribe Varatril if you are willing to take and completely understand the risks. The dermatologist suggests that you stop taking Varatril after the reunion, but also knows from previous experience that you will want to continue taking the drug if the psoriasis goes away completely.   In this case, Varatril will cost an additional $20,000. Please imagine you have full health insurance, and you have already met your annual deductible. Your insurance company has a unique program, in which they cover special drugs with a cash payment, called an indemnity (similar to car insurance). Because your physician has indicated that you are a candidate for Varatril, **you receive a $20,000 cash payment from your insurance company. You receive the money regardless of whether you decide to take the Varatril.** You decide whether you spend the cash on the Varatril or instead keep the money for whatever other purposes you may choose. In deciding, please be realistic.

Q245 On the next page you will share your decision.  First, now please write two sentences putting in your own words, (1) your understanding of the treatment options, and (2) your insurance situation, as described above.

________________________________________________________________

End of Block: Psoriasis - VignetteF/Low/Indemnity

# ArthKnee - VignetteG/High/Indemnity

Q64
 You are in generally good health except for pain in your right knee. It only hurts a little or not at all when you are sitting or lying down, but it usually hurts when you walk for more than a block or climb several flights of stairs. The pain goes away with Advil. You enjoy tennis but playing brings on the pain, and limits your ability to play for more than 30-45 minutes at a time. You were a life-long runner, but do not run anymore because it hurts your knee and also your right hip. Taking Advil before playing tennis makes the pain tolerable, but the Advil upsets your stomach, even if you take it with food.   You once damaged your right knee while playing sports, and had arthroscopic surgery on the knee to remove a torn meniscus (a rubbery, C-shaped disc that cushions your knee). An MRI shows some mild arthritic damage in both knees, more on the right than on the left. You also have some arthritic damage in both hips, probably due to your participation in sports.  

Q203 Please refer to the story specifically, and share a sentence describing how you would feel in this medical situation, and your thoughts about your treatment options described so far.

________________________________________________________________

| Page Break |  |
| --- | --- |

Q204 You see an orthopedic surgeon, who finds instability in your right knee, which will require a knee replacement to manage the pain. He recommends that you have the surgery done now since you are otherwise healthy and want to resume running and playing tennis more actively. Because your right knee arthritis is serious, there is no reason to wait for the surgery that is now indicated.   You ask about the recovery time. After surgery, you will be able to walk with a cane within 2-3 weeks. You will be allowed to drive in 4-6 weeks and will be able to return to most activities within 3 months. If your job requires a lot of physical activity, including travel, you may need 3 months of recovery before being fully able to perform your duties. It will be at least 6 months, and can be up to a year, before you regain full strength in your knee.   Complications after surgery are rare (1-2%), but can sometimes be life threatening, if for example you get a blood clot in your leg.   The alternative to surgery is to try a more regular routine of medical management than you have been using, including injections into the knee, strengthening exercises, and different medications for pain.   In this case, surgery will cost an additional $70,000. Please imagine you have full health insurance, and you have already met your annual deductible. Your insurance company has a unique program, in which they cover special drugs with a cash payment, called an indemnity (similar to car insurance). Because your physician has indicated that you are a candidate for surgery, **you receive a $70,000 cash payment from your insurance company. You receive the money regardless of whether you decide to take the surgery**. You decide whether you spend the cash on the surgery or instead keep the money for whatever other purposes you may choose. In deciding, please be realistic.

Q244 On the next page you will share your decision.  First, now please write two sentences putting in your own words, (1) your understanding of the treatment options, and (2) your insurance situation, as described above.

________________________________________________________________

End of Block: ArthKnee - VignetteG/High/Indemnity

# ArthKnee - VignetteG/Low/Indemnity

Q119
 You are in generally good health except for pain in your right knee. It only hurts a little or not at all when you are sitting or lying down, but it usually hurts when you walk for more than a block or climb several flights of stairs. The pain goes away with Advil. You enjoy tennis but playing brings on the pain, and limits your ability to play for more than 30-45 minutes at a time. You were a life-long runner, but do not run anymore because it hurts your knee and also your right hip. Taking Advil before playing tennis makes the pain tolerable, but the Advil upsets your stomach, even if you take it with food.   You once damaged your right knee while playing sports, and had arthroscopic surgery on the knee to remove a torn meniscus (a rubbery, C-shaped disc that cushions your knee). An MRI shows some mild arthritic damage in both knees, more on the right than on the left. You also have some arthritic damage in both hips, probably due to your participation in sports.  

Q205 Please refer to the story specifically, and share a sentence describing how you would feel in this medical situation, and your thoughts about your treatment options described so far.

________________________________________________________________

| Page Break |  |
| --- | --- |

Q206 You see an orthopedic surgeon, who finds no instability in your right knee, but predicts that you will eventually require a knee replacement to manage the pain. He recommends that you have the surgery done now since you are otherwise healthy and want to resume running and playing tennis more actively. He tells you that your right knee arthritis is still mild to moderate, and can’t say for sure how quickly it will get worse.   You ask about the recovery time. After surgery, you will be able to walk with a cane within 2-3 weeks. You will be allowed to drive in 4-6 weeks and will be able to return to most activities within 3 months. If your job requires a lot of physical activity, including travel, you may need 3 months of recovery before being fully able to perform your duties. It will be at least 6 months, and can be up to a year, before you regain full strength in your knee.   Complications after surgery are rare (1-2%), but can sometimes be life threatening, if for example you get a blood clot in your leg.   The alternative to surgery is to try a more regular routine of medical management than you have been using, including injections into the knee, strengthening exercises, and different medications for pain.   In this case, surgery will cost an additional $70,000. Please imagine you have full health insurance, and you have already met your annual deductible. Your insurance company has a unique program, in which they cover special drugs with a cash payment, called an indemnity (similar to car insurance). Because your physician has indicated that you are a candidate for surgery, **you receive a $70,000 cash payment from your insurance company. You receive the money regardless of whether you decide to take the surgery**. You decide whether you spend the cash on the surgery or instead keep the money for whatever other purposes you may choose. In deciding, please be realistic.

Q243 On the next page you will share your decision.  First, now please write two sentences putting in your own words, (1) your understanding of the treatment options, and (2) your insurance situation, as described above.

________________________________________________________________

End of Block: ArthKnee - VignetteG/Low/Indemnity

# ArthKnee - VignetteG/High/Traditional

Q120
 You are in generally good health except for pain in your right knee. It only hurts a little or not at all when you are sitting or lying down, but it usually hurts when you walk for more than a block or climb several flights of stairs. The pain goes away with Advil. You enjoy tennis but playing brings on the pain, and limits your ability to play for more than 30-45 minutes at a time. You were a life-long runner, but do not run anymore because it hurts your knee and also your right hip. Taking Advil before playing tennis makes the pain tolerable, but the Advil upsets your stomach, even if you take it with food.   You once damaged your right knee while playing sports, and had arthroscopic surgery on the knee to remove a torn meniscus (a rubbery, C-shaped disc that cushions your knee). An MRI shows some mild arthritic damage in both knees, more on the right than on the left. You also have some arthritic damage in both hips, probably due to your participation in sports.

Q207 Please refer to the story specifically, and share a sentence describing how you would feel in this medical situation, and your thoughts about your treatment options described so far.

________________________________________________________________

| Page Break |  |
| --- | --- |

Q208 You see an orthopedic surgeon, who finds instability in your right knee, which will require a knee replacement to manage the pain. He recommends that you have the surgery done now since you are otherwise healthy and want to resume running and playing tennis more actively. Because your right knee arthritis is serious, there is no reason to wait for the surgery that is now indicated.   You ask about the recovery time. After surgery, you will be able to walk with a cane within 2-3 weeks. You will be allowed to drive in 4-6 weeks and will be able to return to most activities within 3 months. If your job requires a lot of physical activity, including travel, you may need 3 months of recovery before being fully able to perform your duties. It will be at least 6 months, and can be up to a year, before you regain full strength in your knee.   Complications after surgery are rare (1-2%), but can sometimes be life threatening, if for example you get a blood clot in your leg.   The alternative to surgery is to try a more regular routine of medical management than you have been using, including injections into the knee, strengthening exercises, and different medications for pain.   In this case, surgery will cost an additional $70,000. Please imagine **you have full health insurance**, and you have already met your annual deductible, so there is no additional cost to you out of pocket, if you choose to take the surgery treatment. In deciding, please be realistic.

Q242 On the next page you will share your decision.  First, now please write two sentences putting in your own words, (1) your understanding of the treatment options, and (2) your insurance situation, as described above.

________________________________________________________________

End of Block: ArthKnee - VignetteG/High/Traditional

# ArthKnee - VignetteG/Low/Traditional

Q121
 You are in generally good health except for pain in your right knee. It only hurts a little or not at all when you are sitting or lying down, but it usually hurts when you walk for more than a block or climb several flights of stairs. The pain goes away with Advil. You enjoy tennis but playing brings on the pain, and limits your ability to play for more than 30-45 minutes at a time. You were a life-long runner, but do not run anymore because it hurts your knee and also your right hip. Taking Advil before playing tennis makes the pain tolerable, but the Advil upsets your stomach, even if you take it with food.   You once damaged your right knee while playing sports, and had arthroscopic surgery on the knee to remove a torn meniscus (a rubbery, C-shaped disc that cushions your knee). An MRI shows some mild arthritic damage in both knees, more on the right than on the left. You also have some arthritic damage in both hips, probably due to your participation in sports.  

Q209 Please refer to the story specifically, and share a sentence describing how you would feel in this medical situation, and your thoughts about your treatment options described so far.

________________________________________________________________

| Page Break |  |
| --- | --- |

Q210 You see an orthopedic surgeon, who finds no instability in your right knee, but predicts that you will eventually require a knee replacement to manage the pain. He recommends that you have the surgery done now since you are otherwise healthy and want to resume running and playing tennis more actively. He tells you that your right knee arthritis is still mild to moderate, and can’t say for sure how quickly it will get worse. You ask about the recovery time. After surgery, you will be able to walk with a cane within 2-3 weeks. You will be allowed to drive in 4-6 weeks and will be able to return to most activities within 3 months. If your job requires a lot of physical activity, including travel, you may need 3 months of recovery before being fully able to perform your duties. It will be at least 6 months, and can be up to a year, before you regain full strength in your knee. Complications after surgery are rare (1-2%), but can sometimes be life threatening, if for example you get a blood clot in your leg. The alternative to surgery is to try a more regular routine of medical management than you have been using, including injections into the knee, strengthening exercises, and different medications for pain. In this case, surgery will cost an additional $70,000. Please imagine **you have full health insurance**, and you have already met your annual deductible, so there is no additional cost to you out of pocket, if you choose to take the surgery treatment. In deciding, please be realistic.

Q241 On the next page you will share your decision.  First, now please write two sentences putting in your own words, (1) your understanding of the treatment options, and (2) your insurance situation, as described above.

________________________________________________________________

End of Block: ArthKnee - VignetteG/Low/Traditional

# ArthKnee - VignetteG/High/Uninsured

Q122
 You are in generally good health except for pain in your right knee. It only hurts a little or not at all when you are sitting or lying down, but it usually hurts when you walk for more than a block or climb several flights of stairs. The pain goes away with Advil. You enjoy tennis but playing brings on the pain, and limits your ability to play for more than 30-45 minutes at a time. You were a life-long runner, but do not run anymore because it hurts your knee and also your right hip. Taking Advil before playing tennis makes the pain tolerable, but the Advil upsets your stomach, even if you take it with food.   You once damaged your right knee while playing sports, and had arthroscopic surgery on the knee to remove a torn meniscus (a rubbery, C-shaped disc that cushions your knee). An MRI shows some mild arthritic damage in both knees, more on the right than on the left. You also have some arthritic damage in both hips, probably due to your participation in sports.  

Q211 Please refer to the story specifically, and share a sentence describing how you would feel in this medical situation, and your thoughts about your treatment options described so far.

________________________________________________________________

| Page Break |  |
| --- | --- |

Q212 You see an orthopedic surgeon, who finds instability in your right knee, which will require a knee replacement to manage the pain. He recommends that you have the surgery done now since you are otherwise healthy and want to resume running and playing tennis more actively. Because your right knee arthritis is serious, there is no reason to wait for the surgery that is now indicated. You ask about the recovery time. After surgery, you will be able to walk with a cane within 2-3 weeks. You will be allowed to drive in 4-6 weeks and will be able to return to most activities within 3 months. If your job requires a lot of physical activity, including travel, you may need 3 months of recovery before being fully able to perform your duties. It will be at least 6 months, and can be up to a year, before you regain full strength in your knee. Complications after surgery are rare (1-2%), but can sometimes be life threatening, if for example you get a blood clot in your leg. The alternative to surgery is to try a more regular routine of medical management than you have been using, including injections into the knee, strengthening exercises, and different medications for pain. In this case, surgery will cost an additional $70,000. Please imagine that **you currently do not have health insurance**, however you do have the same amount of wealth and credit that you actually have in the real world (the amount you provided in your answer above). So, only if you have enough money available, you can pay for the surgery yourself. Think back to your answer to the purchasing power above. If you do have that much purchasing power, please consider however, other things you may prefer to do with the money. Please be realistic.

Q240 On the next page you will share your decision.  First, now please write two sentences putting in your own words, (1) your understanding of the treatment options, and (2) your insurance situation, as described above.

________________________________________________________________

End of Block: ArthKnee - VignetteG/High/Uninsured

# ArthKnee - VignetteG/Low/Uninsured

Q123
 You are in generally good health except for pain in your right knee. It only hurts a little or not at all when you are sitting or lying down, but it usually hurts when you walk for more than a block or climb several flights of stairs. The pain goes away with Advil. You enjoy tennis but playing brings on the pain, and limits your ability to play for more than 30-45 minutes at a time. You were a life-long runner, but do not run anymore because it hurts your knee and also your right hip. Taking Advil before playing tennis makes the pain tolerable, but the Advil upsets your stomach, even if you take it with food.   You once damaged your right knee while playing sports, and had arthroscopic surgery on the knee to remove a torn meniscus (a rubbery, C-shaped disc that cushions your knee). An MRI shows some mild arthritic damage in both knees, more on the right than on the left. You also have some arthritic damage in both hips, probably due to your participation in sports.  

Q213 Please refer to the story specifically, and share a sentence describing how you would feel in this medical situation, and your thoughts about your treatment options described so far.

________________________________________________________________

| Page Break |  |
| --- | --- |

Q214 You see an orthopedic surgeon, who finds no instability in your right knee, but predicts that you will eventually require a knee replacement to manage the pain. He recommends that you have the surgery done now since you are otherwise healthy and want to resume running and playing tennis more actively. He tells you that your right knee arthritis is still mild to moderate, and can’t say for sure how quickly it will get worse.   You ask about the recovery time. After surgery, you will be able to walk with a cane within 2-3 weeks. You will be allowed to drive in 4-6 weeks and will be able to return to most activities within 3 months. If your job requires a lot of physical activity, including travel, you may need 3 months of recovery before being fully able to perform your duties. It will be at least 6 months, and can be up to a year, before you regain full strength in your knee.   Complications after surgery are rare (1-2%), but can sometimes be life threatening, if for example you get a blood clot in your leg.   The alternative to surgery is to try a more regular routine of medical management than you have been using, including injections into the knee, strengthening exercises, and different medications for pain.   In this case, surgery will cost an additional $70,000. Please imagine that **you currently do not have health insurance,** however you do have the same amount of wealth and credit that you actually have in the real world (the amount you provided in your answer above). So, only if you have enough money available, you can pay for the surgery yourself. Think back to your answer to the purchasing power above. If you do have that much purchasing power, please consider however, other things you may prefer to do with the money. Please be realistic.

Q239 On the next page you will share your decision.  First, now please write two sentences putting in your own words, (1) your understanding of the treatment options, and (2) your insurance situation, as described above.

________________________________________________________________

End of Block: ArthKnee - VignetteG/Low/Uninsured

# ArthSpine - VignetteH/High/Indemnity

Q65
 You are an office worker who has just started having lower back pain. You had a similar problem 4 years ago, when you hurt your lower back lifting a heavy box at work. At the time, you saw your primary care provider (PCP) who prescribed pain medications and bed rest. The pain gradually went away over two weeks. Your PCP had referred you for physical therapy, and gave you a series of lower back exercises to begin when the pain was less severe, but you did not follow through with either.   Four weeks ago, while working in your yard at home, you felt a sharp pain in your lower back in the same area as last time. You saw your PCP, who examined you, found no abnormalities other than tightness and soreness of muscles in your back, and again advised bed rest and pain medication. However, after 4 weeks, you still had severe pain and difficulty walking or sitting. You had many side effects from the pain medications, most notably, severe constipation. You also had to return to work after two weeks, even though it still hurt badly, because you had used up your sick days.   Now, after 3 months with continued pain and stiffness, you are getting more and more discouraged, because you continue to have difficulty with daily activities.

Q215 Please refer to the story specifically, and share a sentence describing how you would feel in this medical situation, and your thoughts about your treatment options described so far.

________________________________________________________________

| Page Break |  |
| --- | --- |

Q216 You ask your PCP what else can be done. She orders an MRI, and this test reveals some abnormalities in your lower spine in the same area as your pain. Given these findings, she refers you to a neurosurgeon for consideration of possible back surgery.   The neurosurgeon evaluates you, tells you that the pain is most likely due to the abnormalities seen on the MRI, and that these are leading to some instability in your back. He suggests surgery to correct the instability, and says that with newer techniques, and in your circumstance, this can be done as outpatient surgery with a short recovery time.   The neurosurgeon says that the new kind of surgery relieves almost all the pain and disability in about 2/3 of cases like yours. The results can vary a lot between patients, but still, these results are much better than with medications only. The alternative is more intense medical treatment, including a regular and continued program of physical therapy, back exercises, and weight loss. However, many patients have trouble sticking with such an intense routine, so they do not recover. Complications from surgery, including infections or bleeding, are rare, occurring in only 1-3% of patients. Physical therapy will be recommended after surgery.   In this case, spinal surgery will cost an additional $85,000. Please imagine you have full health insurance, and you have already met your annual deductible. Your insurance company has a unique program, in which they cover special drugs with a cash payment, called an indemnity (similar to car insurance). Because your physician has indicated that you are a candidate for spinal surgery, **you receive a $85,000 cash payment from your insurance company. You receive the money regardless of whether you decide to take the spinal surgery.** You decide whether you spend the cash on the spinal surgery or instead keep the money for whatever other purposes you may choose. In deciding, please be realistic.

Q238 On the next page you will share your decision.  First, now please write two sentences putting in your own words, (1) your understanding of the treatment options, and (2) your insurance situation, as described above.

________________________________________________________________

End of Block: ArthSpine - VignetteH/High/Indemnity

# ArthSpine - VignetteH/High/Traditional

Q125
 You are an office worker who has just started having lower back pain. You had a similar problem 4 years ago, when you hurt your lower back lifting a heavy box at work. At the time, you saw your primary care provider (PCP) who prescribed pain medications and bed rest. The pain gradually went away over two weeks. Your PCP had referred you for physical therapy, and gave you a series of lower back exercises to begin when the pain was less severe, but you did not follow through with either.   Four weeks ago, while working in your yard at home, you felt a sharp pain in your lower back in the same area as last time. You saw your PCP, who examined you, found no abnormalities other than tightness and soreness of muscles in your back, and again advised bed rest and pain medication. However, after 4 weeks, you still had severe pain and difficulty walking or sitting. You had many side effects from the pain medications, most notably, severe constipation. You also had to return to work after two weeks, even though it still hurt badly, because you had used up your sick days.   Now, after 3 months with continued pain and stiffness, you are getting more and more discouraged, because you continue to have difficulty with daily activities.

Q217 Please refer to the story specifically, and share a sentence describing how you would feel in this medical situation, and your thoughts about your treatment options described so far.

________________________________________________________________

| Page Break |  |
| --- | --- |

Q218 You ask your PCP what else can be done. She orders an MRI, and this test reveals some abnormalities in your lower spine in the same area as your pain. Given these findings, she refers you to a neurosurgeon for consideration of possible back surgery.   The neurosurgeon evaluates you, tells you that the pain is most likely due to the abnormalities seen on the MRI, and that these are leading to some instability in your back. He suggests surgery to correct the instability, and says that with newer techniques, and in your circumstance, this can be done as outpatient surgery with a short recovery time.   The neurosurgeon says that the new kind of surgery relieves almost all the pain and disability in about 2/3 of cases like yours. The results can vary a lot between patients, but still, these results are much better than with medications only. The alternative is more intense medical treatment, including a regular and continued program of physical therapy, back exercises, and weight loss. However, many patients have trouble sticking with such an intense routine, so they do not recover. Complications from surgery, including infections or bleeding, are rare, occurring in only 1-3% of patients. Physical therapy will be recommended after surgery.   In this case, spinal surgery will cost an additional $85,000. Please imagine **you have full health insurance**, and you have already met your annual deductible, so there is no additional cost to you out of pocket, if you choose to take the spinal surgery treatment. In deciding, please be realistic.

Q237 On the next page you will share your decision.  First, now please write two sentences putting in your own words, (1) your understanding of the treatment options, and (2) your insurance situation, as described above.

________________________________________________________________

End of Block: ArthSpine - VignetteH/High/Traditional

# ArthSpine - VignetteH/High/Uninsured

Q127
 You are an office worker who has just started having lower back pain. You had a similar problem 4 years ago, when you hurt your lower back lifting a heavy box at work. At the time, you saw your primary care provider (PCP) who prescribed pain medications and bed rest. The pain gradually went away over two weeks. Your PCP had referred you for physical therapy, and gave you a series of lower back exercises to begin when the pain was less severe, but you did not follow through with either.   Four weeks ago, while working in your yard at home, you felt a sharp pain in your lower back in the same area as last time. You saw your PCP, who examined you, found no abnormalities other than tightness and soreness of muscles in your back, and again advised bed rest and pain medication. However, after 4 weeks, you still had severe pain and difficulty walking or sitting. You had many side effects from the pain medications, most notably, severe constipation. You also had to return to work after two weeks, even though it still hurt badly, because you had used up your sick days.   Now, after 3 months with continued pain and stiffness, you are getting more and more discouraged, because you continue to have difficulty with daily activities.

Q219 Please refer to the story specifically, and share a sentence describing how you would feel in this medical situation, and your thoughts about your treatment options described so far.

________________________________________________________________

| Page Break |  |
| --- | --- |

Q220 You ask your PCP what else can be done. She orders an MRI, and this test reveals some abnormalities in your lower spine in the same area as your pain. Given these findings, she refers you to a neurosurgeon for consideration of possible back surgery.   The neurosurgeon evaluates you, tells you that the pain is most likely due to the abnormalities seen on the MRI, and that these are leading to some instability in your back. He suggests surgery to correct the instability, and says that with newer techniques, and in your circumstance, this can be done as outpatient surgery with a short recovery time.   The neurosurgeon says that the new kind of surgery relieves almost all the pain and disability in about 2/3 of cases like yours. The results can vary a lot between patients, but still, these results are much better than with medications only. The alternative is more intense medical treatment, including a regular and continued program of physical therapy, back exercises, and weight loss. However, many patients have trouble sticking with such an intense routine, so they do not recover. Complications from surgery, including infections or bleeding, are rare, occurring in only 1-3% of patients. Physical therapy will be recommended after surgery.   In this case, spinal surgery will cost an additional $85,000. Please imagine that **you currently do not have health insurance**, however you do have the same amount of wealth and credit that you actually have in the real world (the amount you provided in your answer above). So, only if you have enough money available, you can pay for the spinal surgery yourself. Think back to your answer to the purchasing power above. If you do have that much purchasing power, please consider however, other things you may prefer to do with the money. Please be realistic.

Q236 On the next page you will share your decision.  First, now please write two sentences putting in your own words, (1) your understanding of the treatment options, and (2) your insurance situation, as described above.

________________________________________________________________

End of Block: ArthSpine - VignetteH/High/Uninsured

# ArthSpine - VignetteH/Low/Traditional

Q126
 You are an office worker who has just started having lower back pain. You had a similar problem 4 years ago, when you hurt your lower back lifting a heavy box at work. At the time, you saw your primary care provider (PCP) who prescribed pain medications and bed rest. The pain gradually went away over two weeks. Your PCP had referred you for physical therapy, and gave you a series of lower back exercises to begin when the pain was less severe, but you did not follow through with either.   Four weeks ago, while working in your yard at home, you felt a sharp pain in your lower back in the same area as last time. You saw your PCP, who examined you, found no abnormalities other than tightness and soreness of muscles in your back, and again advised bed rest and pain medication. However, after 4 weeks, you still had severe pain and difficulty walking or sitting. You had many side effects from the pain medications, most notably, severe constipation. You also had to return to work after two weeks, even though it still hurt badly, because you had used up your sick days.   Now, after 3 months with continued pain and stiffness, you are getting more and more discouraged, because you continue to have difficulty with daily activities.  

Q221 Please refer to the story specifically, and share a sentence describing how you would feel in this medical situation, and your thoughts about your treatment options described so far.

________________________________________________________________

| Page Break |  |
| --- | --- |

Q222 You ask your PCP what else can be done. She orders an MRI, and this test reveals some abnormalities in your lower spine in the same area as your pain. Given these findings, she refers you to a neurosurgeon for consideration of possible back surgery.   The neurosurgeon evaluates you, tells you that the pain is most likely due to the abnormalities seen on the MRI, and that these are leading to some instability in your back. He suggests surgery to correct the instability, and says that with newer techniques, and in your circumstance, this can be done as outpatient surgery with a short recovery time.   The neurosurgeon says that surgery relieves almost all the pain and disability in about 1/3 of cases like yours. But the doctor also says that with more intense medical treatment, including a regular and continued program of physical therapy, back exercises, and weight loss, results are about the same as surgery in patients with your condition. Complications from surgery, including infections or bleeding, are rare, occurring in only 1-3% of patients. Physical therapy will be recommended after surgery.   In this case, spinal surgery will cost an additional $85,000. Please imagine **you have full health insurance**, and you have already met your annual deductible, so there is no additional cost to you out of pocket, if you choose to take the spinal surgery treatment. In deciding, please be realistic.

Q235 On the next page you will share your decision.  First, now please write two sentences putting in your own words, (1) your understanding of the treatment options, and (2) your insurance situation, as described above.

________________________________________________________________

End of Block: ArthSpine - VignetteH/Low/Traditional

# ArthSpine - VignetteH/Low/Uninsured

Q128
 You are an office worker who has just started having lower back pain. You had a similar problem 4 years ago, when you hurt your lower back lifting a heavy box at work. At the time, you saw your primary care provider (PCP) who prescribed pain medications and bed rest. The pain gradually went away over two weeks. Your PCP had referred you for physical therapy, and gave you a series of lower back exercises to begin when the pain was less severe, but you did not follow through with either.   Four weeks ago, while working in your yard at home, you felt a sharp pain in your lower back in the same area as last time. You saw your PCP, who examined you, found no abnormalities other than tightness and soreness of muscles in your back, and again advised bed rest and pain medication. However, after 4 weeks, you still had severe pain and difficulty walking or sitting. You had many side effects from the pain medications, most notably, severe constipation. You also had to return to work after two weeks, even though it still hurt badly, because you had used up your sick days.   Now, after 3 months with continued pain and stiffness, you are getting more and more discouraged, because you continue to have difficulty with daily activities.

Q223 Please refer to the story specifically, and share a sentence describing how you would feel in this medical situation, and your thoughts about your treatment options described so far.

________________________________________________________________

| Page Break |  |
| --- | --- |

Q224 You ask your PCP what else can be done. She orders an MRI, and this test reveals some abnormalities in your lower spine in the same area as your pain. Given these findings, she refers you to a neurosurgeon for consideration of possible back surgery. The neurosurgeon evaluates you, tells you that the pain is most likely due to the abnormalities seen on the MRI, and that these are leading to some instability in your back. He suggests surgery to correct the instability, and says that with newer techniques, and in your circumstance, this can be done as outpatient surgery with a short recovery time. The neurosurgeon says that surgery relieves almost all the pain and disability in about 1/3 of cases like yours. But the doctor also says that with more intense medical treatment, including a regular and continued program of physical therapy, back exercises, and weight loss, results are about the same as surgery in patients with your condition. Complications from surgery, including infections or bleeding, are rare, occurring in only 1-3% of patients. Physical therapy will be recommended after surgery. In this case, spinal surgery will cost an additional $85,000. Please imagine that **you currently do not have health insurance**, however you do have the same amount of wealth and credit that you actually have in the real world (the amount you provided in your answer above). So, only if you have enough money available, you can pay for the spinal surgery yourself. Think back to your answer to the purchasing power above. If you do have that much purchasing power, please consider however, other things you may prefer to do with the money. Please be realistic.

Q234 On the next page you will share your decision.  First, now please write two sentences putting in your own words, (1) your understanding of the treatment options, and (2) your insurance situation, as described above.

________________________________________________________________

End of Block: ArthSpine - VignetteH/Low/Uninsured

# ArthSpine - VignetteH/Low/Indemnity

Q124
 You are an office worker who has just started having lower back pain. You had a similar problem 4 years ago, when you hurt your lower back lifting a heavy box at work. At the time, you saw your primary care provider (PCP) who prescribed pain medications and bed rest. The pain gradually went away over two weeks. Your PCP had referred you for physical therapy, and gave you a series of lower back exercises to begin when the pain was less severe, but you did not follow through with either.   Four weeks ago, while working in your yard at home, you felt a sharp pain in your lower back in the same area as last time. You saw your PCP, who examined you, found no abnormalities other than tightness and soreness of muscles in your back, and again advised bed rest and pain medication. However, after 4 weeks, you still had severe pain and difficulty walking or sitting. You had many side effects from the pain medications, most notably, severe constipation. You also had to return to work after two weeks, even though it still hurt badly, because you had used up your sick days.   Now, after 3 months with continued pain and stiffness, you are getting more and more discouraged, because you continue to have difficulty with daily activities.

Q225 Please refer to the story specifically, and share a sentence describing how you would feel in this medical situation, and your thoughts about your treatment options described so far.

________________________________________________________________

| Page Break |  |
| --- | --- |

Q226 You ask your PCP what else can be done. She orders an MRI, and this test reveals some abnormalities in your lower spine in the same area as your pain. Given these findings, she refers you to a neurosurgeon for consideration of possible back surgery.   The neurosurgeon evaluates you, tells you that the pain is most likely due to the abnormalities seen on the MRI, and that these are leading to some instability in your back. He suggests surgery to correct the instability, and says that with newer techniques, and in your circumstance, this can be done as outpatient surgery with a short recovery time.   The neurosurgeon says that surgery relieves almost all the pain and disability in about 1/3 of cases like yours. But the doctor also says that with more intense medical treatment, including a regular and continued program of physical therapy, back exercises, and weight loss, results are about the same as surgery in patients with your condition. Complications from surgery, including infections or bleeding, are rare, occurring in only 1-3% of patients. Physical therapy will be recommended after surgery.   In this case, spinal surgery will cost an additional $85,000. Please imagine you have full health insurance, and you have already met your annual deductible. Your insurance company has a unique program, in which they cover special drugs with a cash payment, called an indemnity (similar to car insurance). Because your physician has indicated that you are a candidate for spinal surgery, **you receive a $85,000 cash payment from your insurance company. You receive the money regardless of whether you decide to take the spinal surgery**. You decide whether you spend the cash on the spinal surgery or instead keep the money for whatever other purposes you may choose. In deciding, please be realistic.

Q231 On the next page you will share your decision.  First, now please write two sentences putting in your own words, (1) your understanding of the treatment options, and (2) your insurance situation, as described above.

________________________________________________________________

End of Block: ArthSpine - VignetteH/Low/Indemnity

# Closing

Q127 In this scenario you just read, considering the alternatives, would you choose ${e://Field/Treatment}?

- Yes (1)
- No (2)

Q128 Please explain your above answer in a sentence.  Why does this seem like the best choice in the situation?

________________________________________________________________

________________________________________________________________

________________________________________________________________

________________________________________________________________

________________________________________________________________

| Page Break |  |
| --- | --- |

| 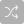 |
| --- |

Q227 Thinking back to the scenario you read, answer this question:  What sort of medical problem were you asked to imagine that you were suffering from?

- Adenocarcinoma of the colon (colon cancer) (1)
- Arthritis of the Spine (2)
- Arthritis of the Knee (3)
- Psoriasis (skin) (4)
- Coronary Artery Disease (heart) (5)
- Gastrointestinal Disease (GERD) (esophagus) (6)
- Age Related Macular Degeneration (AMD) (eyesight) (7)
- Lung Cancer that is spreading to other parts of the body (8)

Q228 Thinking back to the scenario you read, answer this question: Did you have health insurance?

- Yes, in the scenario I had a normal health insurance policy that would pay for the treatment. (1)
- Yes, in the scenario I had an indemnity health insurance policy that paid cash to me regardless of whether I took the treatment. (2)
- No, I did not have insurance in the scenario. (3)

Q40 In the real world, do you currently have health insurance?

- Yes, from employer (own employer or spouse) (1)
- Yes, from Medicaid (2)
- Yes, from individual market (3)
- Yes, from Medicare (4)
- Yes, from some other source (5)
- No, not currently insured. (6)

Q13 Thank you for participating.  If you encountered any problems or have further feedback about this survey, please provide them below.

________________________________________________________________

End of Block: Closing
