# Supplementary material for: Distinguishing moral hazard from access for high-cost healthcare under insurance
Source: PLoS One. 2020 Apr 17;15(4):e0231768. doi: 10.1371/journal.pone.0231768 (PMC7164657; doi:10.1371/journal.pone.0231768)
Supplement: Attachment — Submitted filename: Rebuttal Letter w Letterhead w ctr.docx [file pone.0231768.s015.docx]

1201 E. Speedway

Tucson, AZ 85721-0176

http://www.law.arizona.edu

To:	PLOSOne Editors

Date:	January 18, 2020

Re:	*Distinguishing Moral Hazard from Access in the Context of High-Cost Healthcare*

Thank you for the opportunity to revise our article, which has helped improved its quality and readability. In this letter we provide a point-by-point explanation of how we have incorporated the feedback that we received, and also provide a marked up version of the manuscript as requested. The reviewers’ comments are in *san serif italics* font.

The paper could be improved by adding some discussion of what you are doing into the introduction. It reads as a lit review or state of healthcare but does not give any indication of what the paper will be about.

Thank you; we have added a paragraph at the end of the introduction stating the two contributions of our paper:

“Our study adds to the literature in the following two ways. First, we use indemnities as an alternative theory-driven counterfactual in two vignette-based survey experiments with a high-dollar healthcare setting. Second, our analysis of the experimental outcome provides evidence that most of the healthcare spending from insured consumers would occur even under an indemnity. There is no detectable waste attributable to moral hazard triggered by insurance in the high-dollar healthcare context.”

There are some downsides that need to be addressed in greater detail in the paper. For instance, in the introduction the paper neglects to discuss the idea of negotiated prices. Traditionally insured individuals and uninsured individuals do not only differ in “access” and “moral hazard” components of spending (as in Fig 1) but also differ in what the expected cost of a given healthcare good costs. Insured individuals gain not only the funding for a procedure but also gain the pooled bargaining power of the insurance company. Uninsured individuals in some (most?) cases would face not only the out-of-pocket costs that the insurance company would alternatively pay for but also a second premium for not having bargaining power. The same procedure would likely cost them more to purchase. How would/do indemnities work on this margin? Do the companies that back the indemnities negotiate healthcare prices as traditional insurers do? Citations here are needed as well.

We have added a short paragraph acknowledging these points, including two new citations:

“Aside from laying off risk and providing access to otherwise unaffordable care, insurance has other functions, such as bundling insureds into groups, which have greater market power to negotiate prices (with the threat of excluding a provider from a network), compared to individuals who nominally face undiscounted ‘list’ prices.[cite] On the other hand, uninsured individuals often receive discounted prices based on lower administrative costs and their ability to pay, which can actually then be less than prices offered to insurers.[cite] Although it would be more complicated, an insurer could provide this provider-bargaining function, like a discount card, even if it paid benefits to insureds under an indemnity. (In our experimental framework below, we hold constant the price between indemnities, traditional insurance, and uninsured conditions.)”

Please provide your vignettes in an appendix.

We have provided the entire survey export from Qualtrics, with some reformatting to make it readable and a table of contents to make it navigable.

P11: I like that you provide in-text the wording for the indemnity program. I would also like to see how the uninsured and traditional insurance differs in-text or as a footnote.

Done, in a new footnote (#1).

P12: Clarify for experiment 2 whether this is a between-subject or within-subject design. If a between subject design, please indicate how the 2400 observations are divided between the 3x2x7 treatments. Likewise, for Experiment 1 how were the 613 observations divided between [the] 3x2 treatments?

At the end of the Manipulations subsection, we now explain: “Based on the design described above, Experiment 1 had six conditions (3 insurance types x 2 treatment values), in between-subjects design with equal allocation across all variables and levels thereof. Experiment 2 yielded 48 conditions (3 insurance types x 2 treatment values x 8 clinical scenarios), in between-subjects design with equal allocation across all variables and levels thereof. Manipulations were fully crossed in factorial design to maximize statistical power.”

P12: can you also tell us how participants were paid in MTurk & your other sample? Flat rate? How much? How did you address the concern of bots on MTurk?

For the Mturk sample, we added the following text: “To ensure engagement in the materials and reduce the risk of the work being completed by non-human ‘bots’, we pre-qualified respondents who were successful in at least 90% of prior tasks they had attempted.[46] We also used TurkPrime to block duplicate IP addresses and verify worker country location (United States), and we paid $1.25 to each worker, who (on the median) spent 8.0 minutes to complete the task. … With the above pre-qualification screening and a blinded manual review of qualitative responses, noise such as bots on MTurk are unlikely to be contaminating the final data in our analysis. Due to the randomized control trial design, any noise would bias the analysis towards finding null result. This is not the case as we will see the sharp contrast among responses under two of three different insurance types and both levels of treatment value. Additionally, the estimations are consistent across two experiments, conducted with different populations.”

Such details about payment and recruitment are not available from the SSI pool; those functions are outsourced to the provider.

Figure 2 Note: In the last sentence where you indicate the significant differences or lack of significant differences, please provide the test you are using.

Done.

Please move the table of your main regression analysis to the main text.

Done. See new Table 3.

I would like to see some non-parametric analysis of raw data as seen in figure 2. Mann-Whitney is probably ideal. You are making claims of differences and no differences but are not utilizing the most direct method of analyzing your experimental data and instead base everything off of regressions.

Done. See revised Note for Figure 2, and Table 2.

You attach your stata dta file but not your do file to run your analysis. Please include that in your revision.

Done.

Minor:

Need citation and numbers for claim that high-$ healthcare drives most aggregate healthcare spending in the US

Done (citation 23, Mitchell, et al.).

Some typos remaining in paper (for instance, no brackets around citation #10, #30, or #46)

Done.

You refer to the difference in indemnity and traditional insurance as moral hazard throughout paper but in your tables you call it “waste”. This is somewhat of a disconnect. Why not just call it “M.Hzd” in the parentheses “Insurance (M.Hzd)” to make things more clear?

Done.

P 15: you say “In contrast, we found no moral hazard detectable (at the 0.05 level) in any of our scenarios (0 out of 9). ” this is not exactly true. In table S12 b you have a 5% level significance when controls are excluded. If you want to make this claim in the paper you need to add the qualifier that you are meaning “when controls are included”.

Done; thank you for the close reading.

P19 “rule out the hypothesis that the problem is substantial.” You may want to add a qualifier that this is “in the experimental sample”. You cannot “rule out” that there is a moral hazard in the healthcare sector at large, you can only say that your data suggests that it is not large.

Done.

Overall, we thank you for your close attention to our manuscript. These revisions have substantially improved the paper, and we look forward to publishing with PLOSOne. You can reach me at [**robertson@email.arizona.edu**](mailto:robertson@email.arizona.edu) or 520-621-1289 with any questions.

On behalf of my co-authors, I am sincerely yours,

Christopher T. Robertson, JD, PhD
Professor of Law and Associate Dean for Research & Innovation
