# Supplementary material for: Distinguishing moral hazard from access for high-cost healthcare under insurance
Source: PLoS One. 2020 Apr 17;15(4):e0231768. doi: 10.1371/journal.pone.0231768 (PMC7164657; doi:10.1371/journal.pone.0231768)
Supplement: Attachment — Submitted filename: Response_to_Reviewers.docx [file pone.0231768.s016.docx]

1201 E. Speedway

Tucson, AZ 85721-0176

http://www.law.arizona.edu

To:	PLOSOne Editors

Date:	March 11, 2020

Re:	*Distinguishing Moral Hazard from Access in the Context of High-Cost Healthcare*

Thank you for the opportunity to revise our article a second time. I was particularly happy that you caught the error in the figure! In this letter we provide a point-by-point explanation of how we have incorporated the feedback that we received, and also provide a marked up version of the manuscript as requested. The reviewers’ comments are in *san serif italics* font.

I am ready to accept this paper but there are just a few small edits, that are very minor, but I need to mark the paper as minor revision in order to provide an easy way for you to upload those edits. The direction of these things is not relevant to the scientific rigor of the study but the clarity is needed for the paper.

Yes, thank you for the additional time and attention.

First, Figure 2 and Table 2 are a bit confusing. It appears Indemnity has the highest uptake (consumption) of all three categories in each situation. This would imply a negative moral hazard rather than a positive moral hazard directionally (even if not significant). The labels of the bars and orders of the bars seem to have changed since the first version of the figure, maybe something got mixed up? Table 2 then is hard to interpret the signs of the values in the table (first group minus second group)? Clarifying better what the values in the table are and what is being subtracted from what would be very helpful.

Thank you for catching the error in the figure; the bars were switched making the labels incorrect. In addition to resolving that incongruity, we have also tried to further clarify Table 2.

Second, Table 3 is a bit confusing. The variable "insurance" is self explanatory for the uninsured vs indemnity regression but it is not self explanatory for the indemnity vs the traditional insurance regression. For all of these regression tables, If the variable is indemnity, use that label rather than insurance. If it is "Traditional Insurance" please use that label (and make a separate row to distinguish from indemnity in the other regression model). Added to the confusion from Figure 2/Table 2 these regressions become hard to understand.

We have changed the labels in Table 3, creating the new row as you suggest.

Finally, you say in your response document “Such details about payment and recruitment are not available from the SSI pool; those functions are outsourced to the provider.” This is not necessarily a problem but should just be mentioned. Readers need to know whether they were incentivized in their responses or did it without compensation. I’d recommend contacting your provider and asking them about the incentiveThe paper could be improved by adding some discussion of what you are doing into the introduction. It reads as a lit review or state of healthcare but does not give any indication of what the paper will be about.

We have contacted SSI and received a multiple-paragraph response, which we now include in the supplemental materials verbatim, with one new sentence in the manuscript body referring thereto.

Overall, we thank you for your close attention to our manuscript. We look forward to publishing with PLOSOne. You can reach me at [**robertson@email.arizona.edu**](mailto:robertson@email.arizona.edu) or 520-621-1289 with any questions.

On behalf of my co-authors, I am sincerely yours,

Christopher T. Robertson, JD, PhD
Professor of Law and Associate Dean for Research & Innovation
